# Disturbed intracellular folate homeostasis impairs autophagic flux and increases hepatocytic lipid accumulation

**DOI:** 10.1186/s12915-024-01946-6

**Published:** 2024-07-02

**Authors:** Wan-Yu Chi, Gang-Hui Lee, Ming-Jer Tang, Bing-Hung Chen, Wei-Ling Lin, Tzu-Fun Fu

**Affiliations:** 1 The Institute of Basic Medical Science, College of Medicine, National Cheng Kung University, Tainan, Taiwan; 2 International Center for Wound Repair & Regeneration, National Cheng Kung University, Tainan, Taiwan; 3 Department of Physiology, College of Medicine, National Cheng Kung University, Tainan, Taiwan; 4 Department of Biotechnology, Kaohsiung Medical University, Kaohsiung, Taiwan; 5 Department of Medical Research, Kaohsiung Medical University Hospital, Kaohsiung, Taiwan; 6 Center for Biomarkers and Biotech Drugs, Kaohsiung Medical University, Kaohsiung, Taiwan; 7 Institute of Biomedical Sciences, National Sun Yat-sen University, Kaohsiung, Taiwan; 8 Department of Medical Laboratory Science and Biotechnology, College of Medicine, National Cheng Kung University, No. 1, University Rd, Tainan, 701 Taiwan

**Keywords:** Autophagy, Cathepsin L, Folate deficiency, Lipid metabolism

## Abstract

**Background:**

Metabolic associated fatty liver disease (MAFLD), a prevalent liver disorder affecting one-third of the global population, encompasses a spectrum ranging from fatty liver to severe hepatic steatosis. Both genetic and lifestyle factors, particularly diet and nutrition, contribute to its etiology. Folate deficiency, a frequently encountered type of malnutrition, has been associated with the pathogenesis of MAFLD and shown to impact lipid deposition. However, the underlying mechanisms of this relationship remain incompletely understood. We investigated the impact of disturbed folate-mediated one-carbon metabolism (OCM) on hepatic lipid metabolism both *in vitro* using human hepatoma cells and *in vivo* using transgenic fluorescent zebrafish displaying extent-, stage-, and duration-controllable folate deficiency upon induction.

**Results:**

Disturbed folate-mediated one-carbon metabolism, either by inducing folate deficiency or adding anti-folate drug, compromises autophagy and causes lipid accumulation in liver cells. Disturbed folate status down-regulates cathepsin L, a key enzyme involved in autophagy, through inhibiting mTOR signaling. Interfered mitochondrial biology, including mitochondria relocation and increased fusion-fission dynamics, also occurs in folate-deficient hepatocytes. Folate supplementation effectively mitigated the impaired autophagy and lipid accumulation caused by the inhibition of cathepsin L activity, even when the inhibition was not directly related to folate deficiency.

**Conclusions:**

Disruption of folate-mediated OCM diminishes cathepsin L expression and impedes autophagy via mTOR signaling, leading to lipid accumulation within hepatocytes. These findings underscore the crucial role of folate in modulating autophagic processes and regulating lipid metabolism in the liver.

**Supplementary Information:**

The online version contains supplementary material available at 10.1186/s12915-024-01946-6.

## Background

Metabolic-associated fatty liver disease (MAFLD) is the most prevalent liver disorder worldwide, affecting approximately thirty percent of the global population [1]. It encompasses a spectrum of liver conditions, ranging from simple fatty liver (steatosis) to nonalcoholic steatohepatitis (NASH), which can progress to fibrosis, cirrhosis, and liver cancer, making it a prominent cause of hepatocellular carcinoma (HCC) among non-cirrhotic risk factors in developed countries [2]. Moreover, MAFLD has been linked to an increased risk of all-cause mortality [3]. As a multi-factorial disorder, MAFLD is associated with both genetic predisposition and modifiable environmental risk factors. Obesity, type 2 diabetes mellitus, lifestyle choices, diet, and exercise play crucial roles in the development and progression of MAFLD [4–6]. Understanding the root causes and underlying pathomechanisms of MAFLD becomes imperative for developing effective prophylactic and therapeutic strategies against this disease.

**Fig. 1:**
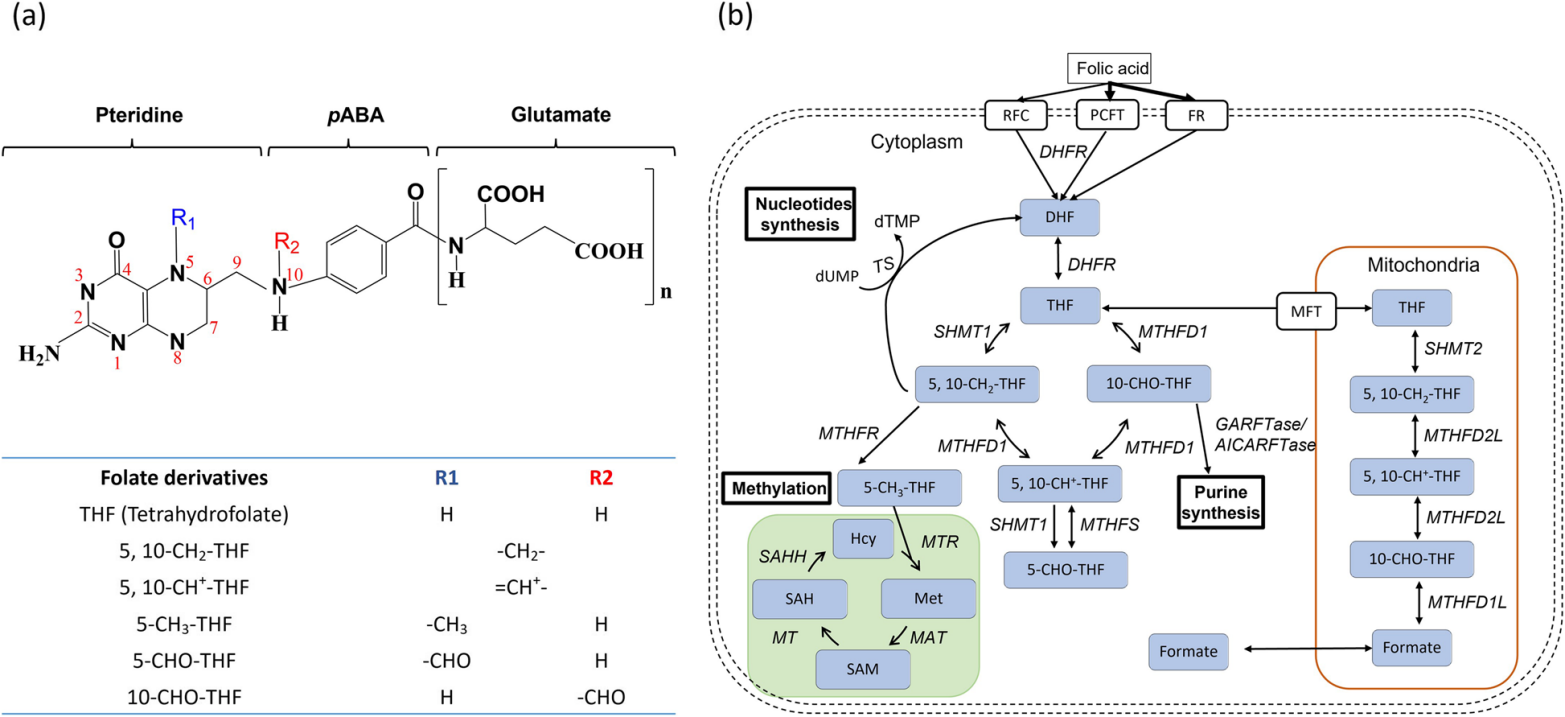
Folate and folate-mediated one-carbon metabolism (OCM). **a** Folate comprises a pteridine ring and a p-aminobenzoic acid with polyglutamyl residues attached to the carboxyl group of the benzene ring. The one-carbon unit attached to the N5 and/or N10 positions of the pteridine ring is in the oxidation states of either formate, formaldehyde, or methanol. **b** Folate, through folate-mediated one-carbon metabolism (OCM), provides its one-carbon unit for purine and thymidylate biosynthesis, amino acid metabolism, S-adenosylmethionine formation, and redox homeostasis. Abbreviations: DHFR, dihydrofolate reductase; MTHFD1, methylenetetrahydrofolate dehydrogenase 1; SHMT1, cytosolic serine hydroxymethyltransferase; MTHFS, 5,10-methenyltetrahydrofolate synthetase; MTHFR, methylenetetrahydrofolate reductase; TS, thymidylate synthase; MTR, 5-methyltetrahydrofolate-homocysteine methyltransferase; MAT, methionine adenosyl transferase; MT, methyltransferase; SAHH, S-adenosylhomocysteine hydrolase; SHMT2, mitochondrial serine hydroxymethyltransferase-2; MTHFD1L, mitochondrial 10-formy-THF synthetase; MTHFD2/2L, mitochondrial bi-functional enzyme (5,10-methylene-THF dehydrogenase/5,10-methenyl-THF cyclohydrolase); MFT, mitochondrial folate transporter; RFC, reduced folate carrier; PCFT, proton-couple folate transporter; FR, folate receptor

The deficiency of folate, a water-soluble vitamin B, has been shown to impact lipid metabolism. In the context of Metabolic-Associated Fatty Liver Disease (MAFLD), fat accumulation in the liver is a key feature. Liver is the primary organ responsible for lipid metabolism and folate storage. Notably, serum folate levels have been associated with lipid profiles, highlighting the significance of folate in hepatic lipid metabolism [7]. Folate serves as a one-carbon carrier in folate-mediated one-carbon metabolism (OCM), contributing to the biosynthesis and metabolism of nucleic acids, proteins, amino acids, neurotransmitters, and certain vitamins (Fig. 1). Consequently, folate plays a vital role in rapidly proliferating cells and growing tissues, such as the fetus and cancer cells. Folate is also essential for forming S-adenosylmethionine, the primary methyl donor for numerous intracellular methylation reactions involving macromolecules like DNA/RNA, proteins, and lipids. Thus, folate plays a crucial role in the epigenetic control of gene activity and metabolic programming [8]. The biological importance of folate, coupled with the widespread accessibility of folate supplements to the general public, has rendered this vitamin a potent tool for promoting public well-being [9]. Equipped with the most abundant folate, folate binding proteins and folate enzymes among tissues, the liver serves as the primary organ responsible for storing and releasing folate into the bloodstream as needed. Despite the significant role of folate in lipid metabolic homeostasis and liver function, the underlying mechanisms by which folate influences these processes remain incompletely understood.

Disturbed folate-mediated one-carbon metabolism, including both impaired folate homeostasis and imbalanced folate content/composition, has been shown to disrupt autophagy. Autophagy is a complex cellular process in which cells break down and recycle damaged cellular components or macromolecules, including lipids, in response to cellular stress and nutrient deprivation [10]. Dysregulation of autophagy has been linked to the pathogenesis of various diseases, including metabolic disorders [11]. Moreover, autophagy has been implicated in mediating the crosstalk between innate immune responses and MAFLD [12]. A specific type of autophagy, called lipophagy, involves the selective degradation of lipid droplets, preventing the excessive buildup of lipids within cells [13, 14]. Various factors, particularly environmental stressors and nutrient deprivation such as folate deficiency, can lead to dysfunctional autophagy [15, 16]. Additionally, folate supplementation has been reported to protect cells from autophagic dysfunction induced by accumulated homocysteine, a metabolic intermediate in folate-mediated OCM [17]. Currently, the exact mechanisms underlying the interplay among intracellular folate status, autophagic control and its impact on hepatic lipid metabolism remain elusive.

We have observed both apparent lipid accumulation and hepatomegaly in the liver of zebrafish displaying folate deficiency (FD). Previously, we have established a fluorescent double transgenic zebrafish line, Tg(*lfabp*:mCherry/*hsp*:eGFP-γGH), which consistently exhibits red fluorescence in liver and reduced intracellular folate content throughout the entire body in an extent-, stage-and duration-controllable manner upon heat-shock induction [18]. Contrary to our initial hypothesis, the observed hepatomegaly in these FD fish was not attributed to nucleotide depletion or lipid accumulation. Instead, it emerged as a consequence of the collaborative interplay among inflammation, necroptosis, and the YAP signaling pathway [19]. In the present study, we delved into the potential mechanism underlying FD-induced hepatic lipid accumulation and discovered that impaired autophagy, due to down-regulated cathepsin L, played a crucial role in this phenomenon. Furthermore, we examined and discussed the effectiveness of folate supplementation in preventing hepatic lipid accumulation induced by factors other than folate deficiency.

## Results

### Lipid accumulation was observed in the liver of FD zebrafish

**Fig. 2:**
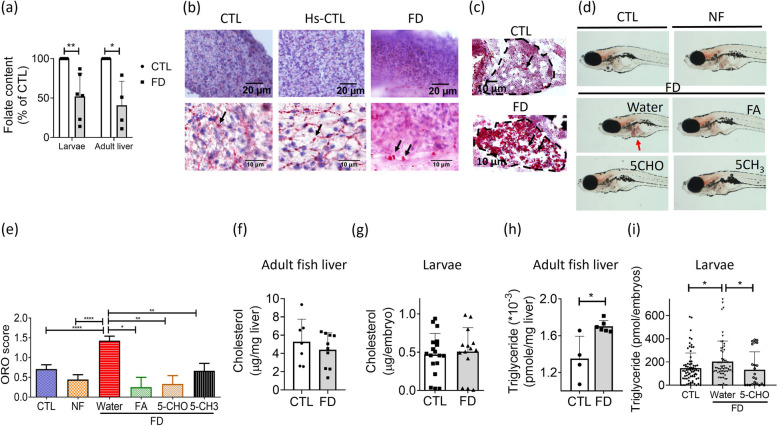
Increased lipid accumulation was found in FD zebrafish liver. The transgenic larvae and adult fish were induced for FD and examined for their hepatic lipid deposition. **a** The liver of larvae of 11 dpf and adult fish after completing FD-induction protocols were collected and subjected to microbiology assay. Data were collected from 6 and 4 independent experiments for larval liver and adult fish liver, respectively. **b**-**c** The cryosections prepared from adult fish liver (**b**) and whole larvae (**c**) were examined for lipid deposition (black arrows) with Oil Red O staining. **d**-**e** The hepatic lipid accumulation (red arrow) in larvae at 11 dpf, with/without FA, 5-CHO-THF, and 5-CH_3_-THF supplementation, were stained with Oil Red O (red arrow) (**d**) and scored (**e**) following the criteria based on the percentage of intensely stained area occupied in larval liver: less than 30% (1), between 30% to 70% (2), or more than 70% (3). Those showed no significant staining signal was scored (0). Data were collected from at least 3 independent experiments. **f**-**i** Hepatic cholesterol (**f**-**g**) and triglyceride (**h**-**i**) in adult fish liver (**f** and **h**) and larvae at 11 dpf (**g** and **i**) were measured with colorimetric/fluorometric Assay. Significant increase was found for the triglyceride levels in both adult fish liver and larval liver. Supplementing with 5-CHO-THF effectively prevented the increase of triglyceride in FD larvae. All the data presented are the averages of at least three independent trials with 10-40 larvae or 4-10 adult fish livers for each group. CTL, control (Tg-GGH/LR larvae without FD); FD, folate deficiency; FA, folic acid; 5-CHO, 5-formyl-THF; 5-CH_3,_ 5-methyl-THF. Statistical data are shown in mean ± SEM. * *p*<0.05, **, *p* <0.01; ****, *p*<0.0001

To examine whether FD affect hepatic lipid metabolism and deposition, the whole larvae and the livers of adult zebrafish were subjected to folate content measurement and histopathological examination with Oil red O staining. Decreased folate content was apparent in Tg(*lfabp*:mCherry/*hsp*:eGFP-γGH) larvae (FD larvae) and the liver of Tg(*lfabp*:mCherry/*hsp*:eGFP-γGH) adult fish (FD adult fish), confirming the successful induction of FD (Fig. 2a). The signals of Oil red O staining, indicating lipid deposition, were apparent in the cryo-sections prepared from the liver of FD adult fish and larvae (Fig. 2b and c). Observing the whole larvae under a light dissecting microscope also revealed an increased signal of Oil red O staining in FD larval liver, which was prevented by supplementing with folic acid, 5-CHO-THF or 5-CH_3_-THF, confirming the causal-link between FD and increased hepatic lipid accumulation (Fig. 2d and e). Both adult fish liver and whole larvae of 11dpf were also subjected to lipid content analysis. No appreciable difference in the total cholesterol levels was found in either FD adult fish liver or FD larvae (Fig. 2f and g). Contrarily, the triglyceride levels were significantly elevated both in the liver of FD adult fish and FD larvae, which was rescued by adding 5-CHO-THF to embryo water, further supporting the causal role of FD for triglyceride accumulation (Fig. 2h and i). These results show that FD disturbs lipid metabolism and increases lipid deposition in liver.

### Increased lipid content and hepato-pathological marker were found in FD hepatoma cells

**Fig 3:**
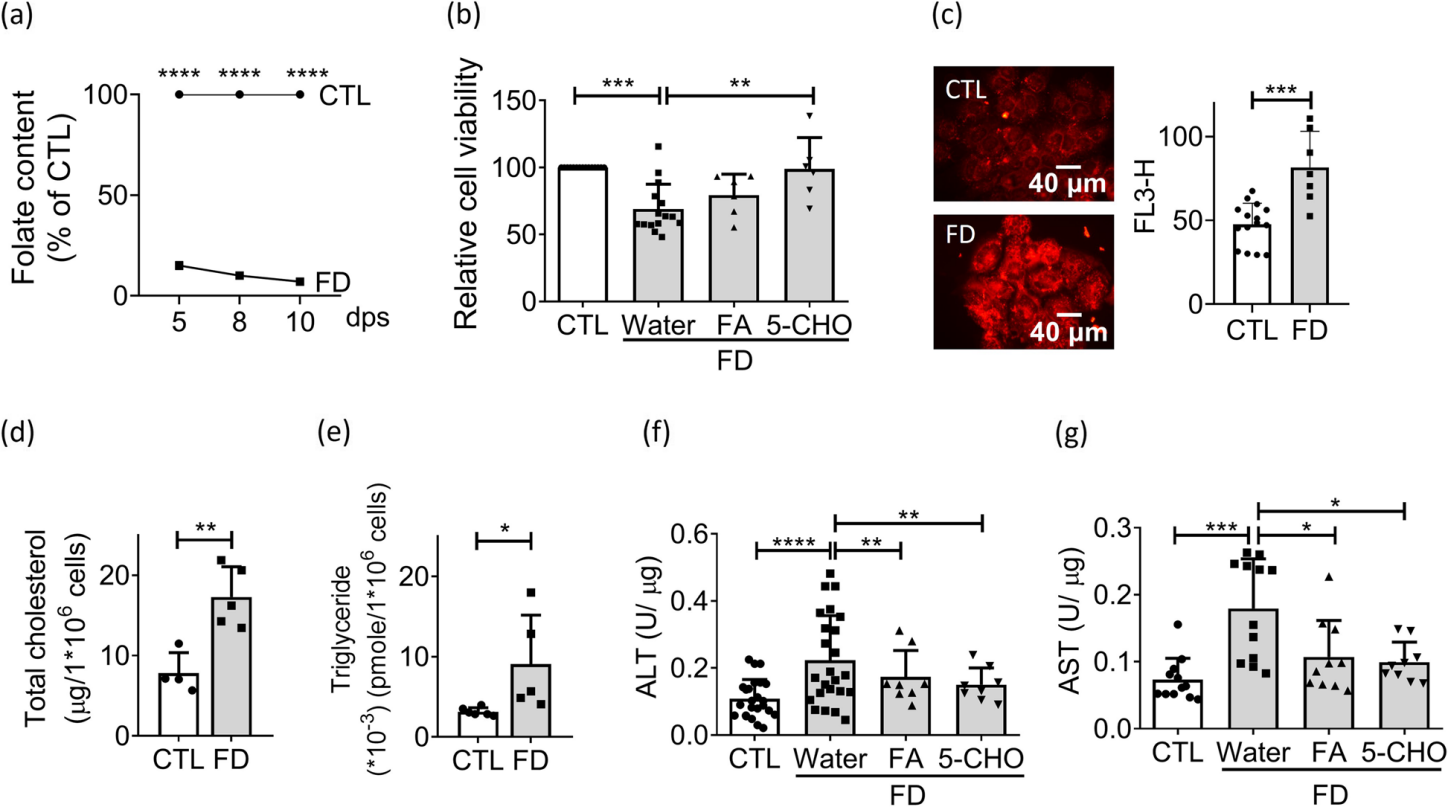
Increased lipid deposition was found in FD Huh7 cells. Huh7 cells were cultivated in FD medium and examined for cell viability and lipid content. **a** Huh7 cells cultivated in CTL (0.004 g/L folic acid) or folate-deficient (FD) medium were analyzed with microbiology assay for folate content. A more than 90% depletion of intracellular folate content was established at 10 day-post-seeding (dps) for the cells growing in FD medium. Presented are data collected from 3 and 4 independent experiments at 5 dps, and 8 and 10 dps, respectively. **b** Huh7 cells were cultivated in CTL or FD medium for 7 days (dps) before adding FA or 5-CHO-THF for rescue. Cell viability was examined on 8 dps. The decreased cell viability due to FD was successfully rescued by the presence of FA or 5-CHO-THF. Data were collected from at least 6 independent experiments. **c** Cells were stained with Nile red and analyzed for lipid content with direct visual inspection with fluorescence microscopy (left) and quantified by flow cytometry (right). Scale bars = 40 µm. Data were collected from at least 7 independent experiments. **d**-**e** The cholesterol (**d**) and triglyceride (**e**) levels of Huh7 cells at 8 dps was measured with colorimetric/fluorometric Assay. Data were collected from at least 6 independent experiments. **f**-**g** Cells cultivated in FD medium with/without FA or 5-CHO-THF supplementation were examined for ALT (**f**) and AST (**g**) content. The increase of both ALT and AST was effectively alleviated by supplementing with FA or 5-CHO-THF. Presented are the averaged results of at least 8 independent trials. CTL, control (cells without FD); FD, folate deficiency. Statistical data are shown in mean ± SEM. * *p*<0.05, **, *p* <0.01; ***, *p*<0.001, ****, *p*<0.0001

For subsequent investigation in depth, FD *in vitro* model was established by cultivating Huh7 cells, an immortal human hepatoma cell line, in FD medium and examined for intracellular folate levels, cell viability and lipid content. A time-dependent decrease in the intracellular folate content was found, with a close to 90% depletion achieved 8 days after cultivation in FD medium (Fig. 3a). Decreased viability was also observed when cells were grown in FD medium for 8 days, which was effectively prevented by 5-CHO-THF supplementation, confirming the causal role of FD (Fig. 3b). Both the fluorescence cell imaging and flow cytometric analysis revealed stronger fluorescence intensities of Nile Red staining in the cells cultivated in FD medium, as compared to those grown in the control medium (Fig 3c). These observations indicate an increased lipid accumulation in the FD cells. The levels of both total cholesterol and triglyceride produced by the FD cells were significantly higher than those in the CTL medium (Fig. 3d and e). Similar results were observed for the total activities of aspartate aminotransferase (AST) and alanine aminotransferase (ALT), the markers for liver injury, in which significantly higher enzymatic activities were detected in the FD cells than in the control cells (Fig. 3f and g). Furthermore, folate supplementation effectively prevented the elevation of cellular ALT and AST activities in response to FD. These results further support the notions that FD increases lipid accumulation in liver cells and implies an elevated risk of liver injury.

## An activated but compromised autophagic pathway was found in FD Huh7 cells

**Fig. 4:**
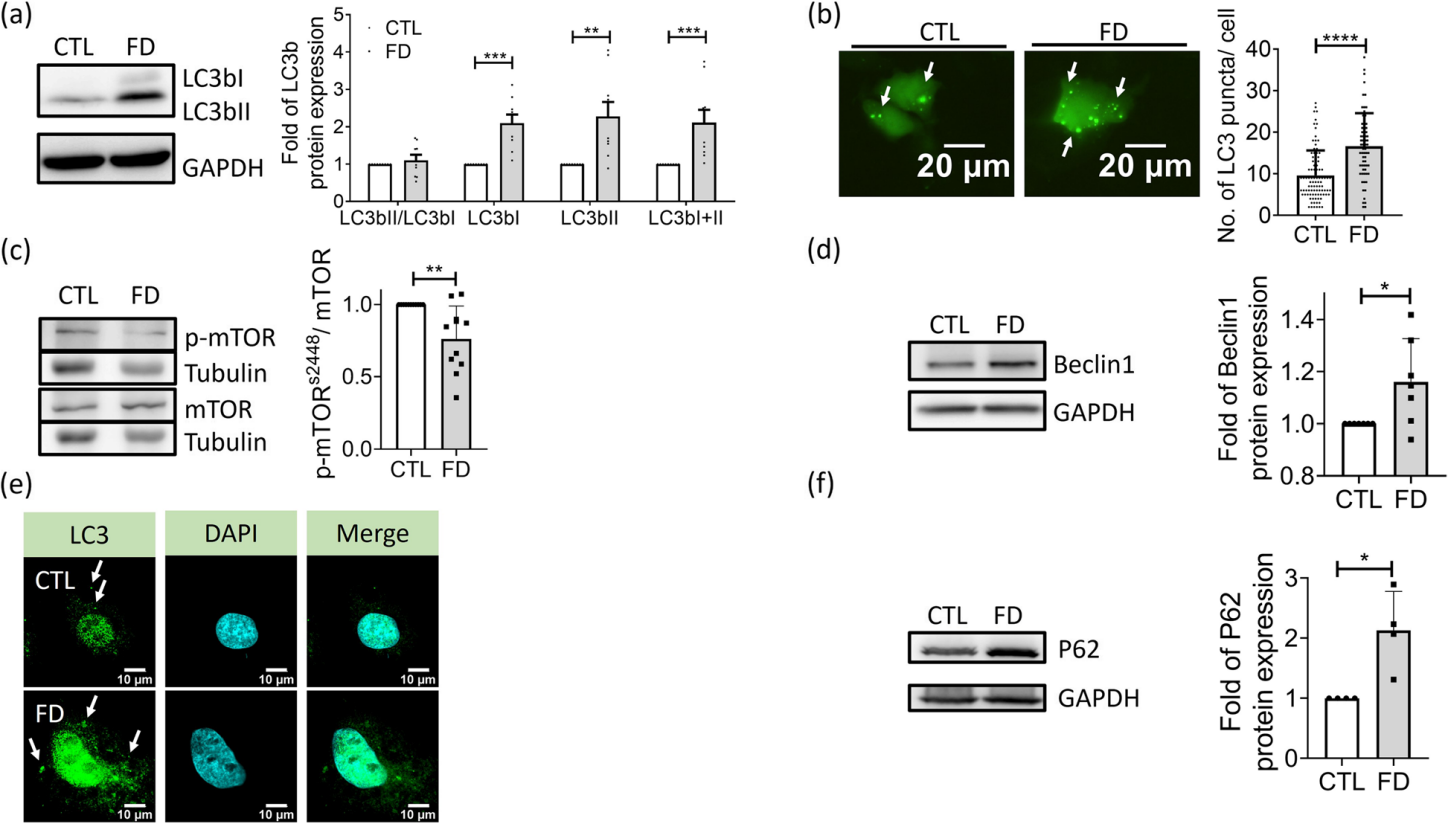
Impaired autophagic flux was found in FD Huh7 cells. Huh7 cells cultivated in CTL (0.004 g/L folic acid) or FD medium for 8 days were examined for the occurrence and flow of autophagy. **a** The expression of LC3b, the autophagic marker, was examined with Western blotting (left) and quantified (right). The increase of LC3bI and LC3bII signified an activated autophagy. Presented are the averaged results of 9 independent trials. **b** Cells transfected with plasmids encoding LC3-eGFP were cultivated in CTL (0.004 g/L folic acid) or folate deficient (FD) medium and examined for the green fluorescent puncta, which represent the LC3-containing autophagic vesicles. Scale bars = 20 µm. Data were collected from at least 3 independent experiments with the total sample number of 66-97 for each group. **c**-**d** Cells were examined for the expression of mTOR, p-mTOR^s2448^ and Beclin1. Significantly decreased p-mTOR ^s2448^/mTOR ratio and increased Beclin1 were observed in FD Huh7 cells, suggesting an activation of autophagy and formation of phagophore. Presented are data collected from 11 and 7 independent experiments for mTOR and Beclin1, respectively. **e** Huh7 cells were immuno-stained for LC3 distribution and examined with FV3000 confocal laser scanning microscopy. The increased relocation of dispersed LC3 puncta to cytoplasm (white arrows) was found in FD Huh7 cells. Scale bars = 10 µm. **f** Apparently increased P62 was observed in FD Huh7 cells, indicating an increased autophagosomes. Presented are the averaged results of four independent trials. CTL, control (cells without FD); FD, folate deficiency. Statistical data are shown in mean ± SEM. * *p*<0.05, **, *p* <0.01; ***, *p*<0.001, ****, *p*<0.0001

The lipid accumulation found in FD fish liver has prompted us to survey the expression of genes involved in lipolysis, fatty acid transportation, lipogenesis and adipogenesis. Most of the investigated genes display no significant and consistent alteration in their expression among FD Huh7 cells and the liver of larvae and adult fish, compelling us to examine the integrity of autophagy, the other pathway crucial to lipid catabolism especially in response to nutrient deficiency (Fig. S1). An approximately 2-fold increase was found in the protein levels of LC3bI, LC3bII and total LC3b, the markers of activated autophagy, in FD Huh7 cells, suggesting an activation of autophagic pathway (Fig 4a). Increased number of green fluorescent puncta was also observed in FD Huh7 cells transfected with the plasmids encoding LC3-eGFP, indicating an increased formation of autophagosomes (Fig. 4b). Mechanistic target of rapamycin (mTOR) is a key regulator for maintaining nutrient homeostasis and functions by modulating lysosomal biogenesis and autophagy. Beclin1 is a core component in the lipid-kinase complex involved in autophagosome nucleation. Both the decreased p-mTOR^S2448^/mTOR ratio and increased Beclin 1 found in FD Huh7 cells also indicate an increased autophagic activity (Fig. 4c and d). As expected, confocal microscopy analysis revealed relocation of LC3 puncta to the cytoplasm of FD Huh7 cells, indicating an increased autophagic process (Fig. 4e). However, we also observed the accumulation of P62, an autophagic receptor, suggesting the occurrence of ineffective autophagy, which possibly resulted from a blockage of autophagic flux, in FD liver cells (Fig. 4f). These data indicated that there occurred an activated, but ineffective autophagic process in FD liver cells.

**Fig. 5:**
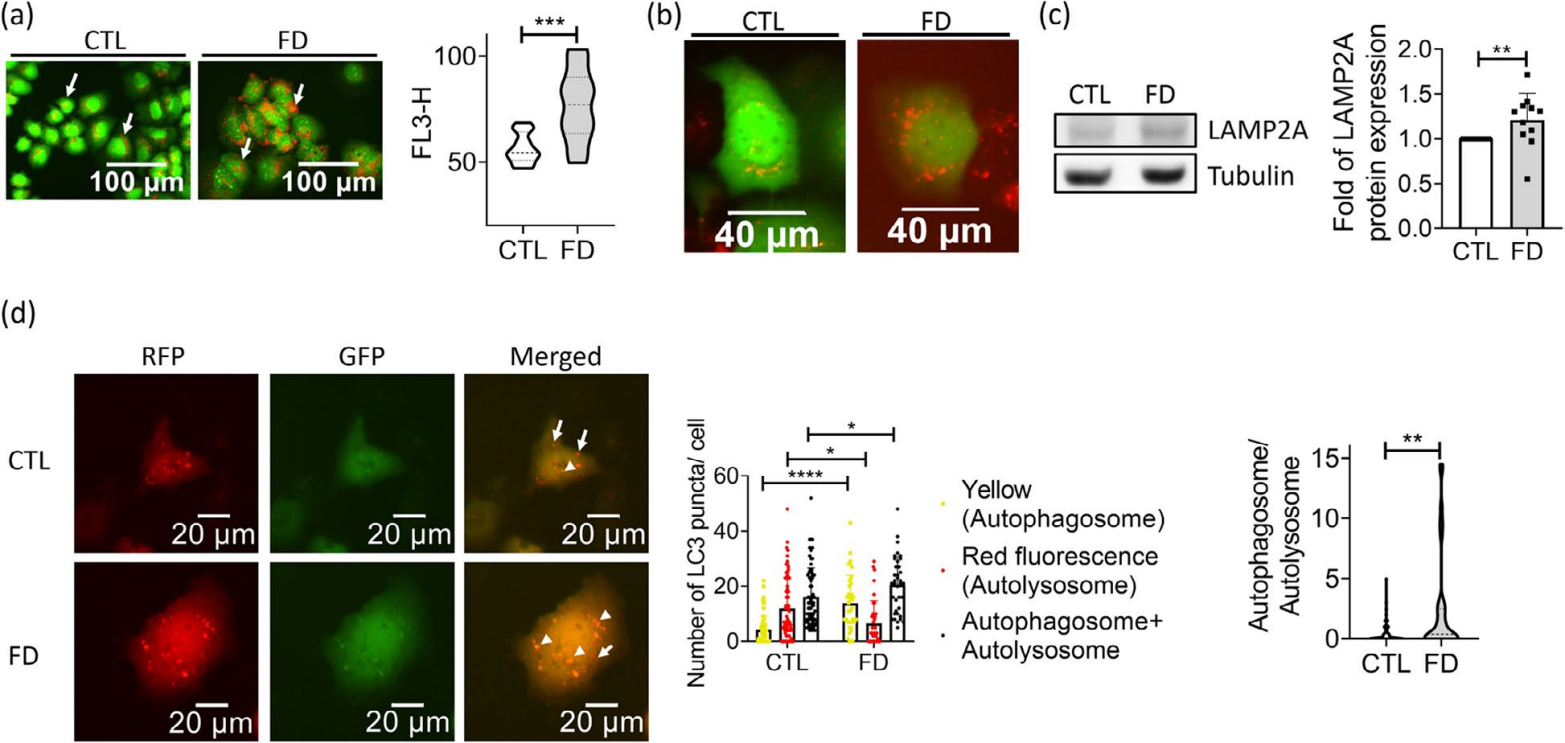
FD caused autophagosomes accumulation in Huh7 cells. Huh7 cells grown in FD medium for 5 days were stained with acridine orange (**a**) and LysoTracker (**b**) for acidic vesicles. Data were collected from at least 3 independent experiments. Cells were also subjected to Western blotting for LAMP2A, a lysosomal membrane protein promoting autophagic flux (**c**). Data were collected from 11 independent experiments. **d** Cells transfected with mRFP-GFP tandem fluorescence-tagged LC3 plasmid were examined for the presence of autophagosome (yellow fluorescent dots) and autolysosome (red fluorescent dots) (left). Increased autophagosomes and decreased autolysosomes were found in FD Huh7 cells (middle), leading to a significantly elevated autophagosome/autolysosome ratio (right). Scale bars = 20 µm. Presented are the averaged results of at least three independent trials. CTL, control (cells without FD); FD, folate deficiency. Statistical data are shown in mean ± SEM. * *p*<0.05, **, *p* <0.01; ***, *p*<0.001, ****, *p*<0.0001

To further examine autophagic flux, cells were stained with acridine orange (AO) and LysoTracker to specifically visualize the acidic vesicular organelles, including lysosomes and autolysosomes. We found that the signals for acridine orange and LysoTracker were both higher in FD cells than in control cells. (Fig 5a and b). The expression of LAMP2A, a lysosomal membrane protein capable of potentiating autophagic flux, was also increased in FD Huh7 cells (Fig. 5c). Cells were transfected with the plasmid encoding mRFP-EGFP tandem fluorescence-tagged LC3 (mRFP-EGFP-LC3) for distinguishing autophagic vesicles, based on the distinct stabilities of EGFP and mRFP fluorescent proteins at different pH, where EGFP is denatured and loses its green fluorescence in acidic environment but mRFP remains intact. Therefore, the presence of yellow fluorescent puncta signals the co-existence of mRFP and EGFP, representing autophagosomes; whereas red fluorescent puncta represent autolysosomes [20]. Our data showed that the numbers of yellow LC3 puncta in FD Huh7 cells were significantly higher than those in control cells, confirming the increased formation of autophagosomes (Fig. 5d). On the other hand, the number of red fluorescent puncta, which represent autolysosomes, were lower in FD cells as compared with that in control. The total number of autophagosome and autolysosome were also higher in FD cells than in control cells, echoing the activation of autophagic process. The significant increase in the ratio between autophagosome and autolysosome found for FD Huh7 cells suggests the blockage in the maturation process of autolysosomes.

## The activity and distribution of mitochondria were perturbed in FD Huh7 cells

**Fig. 6:**
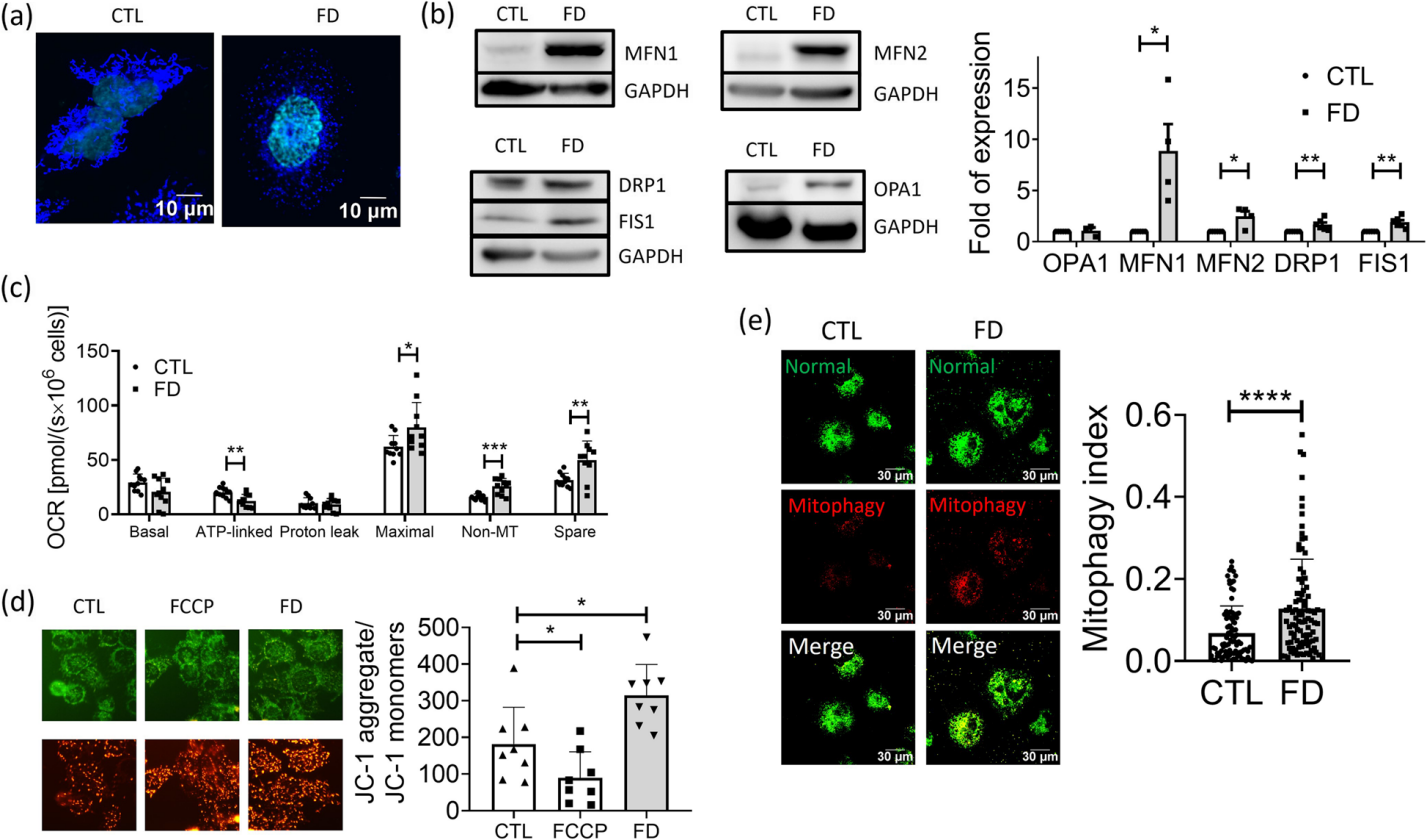
Disturbed mitochondrial activity and distribution were found in FD Huh7 cells. Huh7 cells cultivated in FD medium for 5 days were stained with MitoTracker for mitochondrial morphology (**a**) and subjected to Western blotting for characterizing the expression of mitochondrial fusion-fission proteins (**b**). Significant increase was found for both fusion (MFN1 and MFN2) and fission (DRP1 and FIS1) proteins, suggesting an increased mitochondrial dynamic in FD cells. Data were collected from at least 4 independent experiments. **c** Huh7 cells cultivated in CTL or FD medium for 8 to 9 days were subjected to Oroboros O2k for monitoring mitochondrial activity. Lowered ATP production and elevated non-mitochondria OCR and spare capacity were observed in Huh7 cells. Presented are data collected from at least 9 independent experiments. **d** Cells cultivated in CTL or FD medium for 8 days were stained with JC-1 (5,5',6,6'-tetrachloro-1,1',3,3'-tetraethylbenzimidazolylcarbocyanine iodide) for assessing mitochondrial membrane potential (MMP) changes. The increased ratio between aggregated and monomer mitochondria indicates a higher mitochondrial membrane potential in FD cells. Presented are data collected from at least 8 independent experiments (**e**) Huh7 cells were transfected with mt-Keima, a ratio-metric pH-sensitive fluorescent probe targeting the mitochondrial matrix, at 7 dps. In a neutral environment, mt-Keima predominantly emits a short wavelength (normal, 440 nm), displaying a green mitochondrial signal. Conversely, in an acidic environment, it primarily emits a long wavelength (mitophagy, 586 nm), generating a red lysosomal signal. The 'Mitophagy Index' was determined by calculating the ratio of 586 nm/ 440 nm fluorescence signals in Huh7 cells. Data were collected from at least 3 independent experiments with the total sample number of 90-97 for each group. Statistical data are shown in mean ± SEM. CTL, control (cells without FD); FD, folate deficiency. FCCP, carbonylcyanide-p-trifluoromethoxyphenylhydrazone (a potent uncoupler of oxidative phosphorylation). Statistical data are shown in mean ± SEM. * *p*<0.05, **, *p* <0.01, ****, *p*<0.0001

Mitochondria are the most important organelle for lipid metabolism and energy production, making them essential for maintaining cellular lipid homeostasis and overall energy balance. We examined both the distribution and respiration/bioenergetics of mitochondria in FD cells to evaluate mitochondrial integrity. Unlike in control cells, a perinuclear localization of mitochondria was observed in the cytosol of FD Huh7 cells with MitoTracker staining (Fig. 6a). The expression of mitochondrial proteins related to fusion-fission dynamics, including MFN1, MFN2, DRP1, and FIS1, was significantly increased in FD cells, indicating an increased dynamic shifting (Fig. 6b). Decreased ATP production and increased non-mitochondrial OCR were also observed in FD Huh7 cells (Fig. 6c). Unexpectedly, the mitochondrial membrane potential of FD Huh7 cells was notably elevated compared to that of the control cells (Fig. 6d). Characterization of the mitophagy index revealed enhanced mitophagy in FD cells, as evidenced by increased dispersal and an approximately two-fold elevation (Fig. 6e). The mitophagy index, determined by the ratio of excitation wavelengths (586 nm/440 nm) of the mitochondria-targeted fluorescent protein Keima, indicates the extent of mitophagy. Keima exhibits a pH-dependent spectral excitation property: a short wavelength (440 nm) predominates in a neutral environment, while a long wavelength (586 nm) predominates in an acidic environment. Consequently, Keima displays a high ratio value, depicted in red in a mitophagic environment. Our results suggest that FD significantly impacts mitochondrial function and dynamics.

### Cathepsin L expression was down-regulated in FD liver cells

**Fig. 7:**
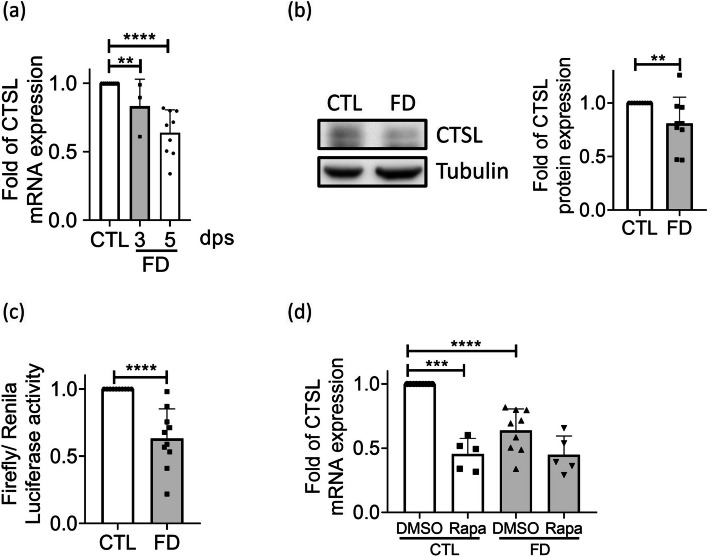
The expression of CTSL was down-regulated in FD Huh7 cells. **a** Huh7 cells cultivated in FD medium were harvested at 3-dps and 5-dps and characterized for cathepsin L expression with real-time PCR. Data were collected from at least 3 independent experiments. **b** The protein levels of cathepsin L, which were examined after cells being cultivated for 5 days, were significantly decreased in those grown in FD medium. The average of nine independent experiments. **c** Huh7 cells transfected with plasmids encompassing zebrafish cathepsin L promoter region (-2500 to +270) were harvested 3 days after transfection and subjected to promoter activity assay. The average of ten independent experiments. **d** Cells cultivated in medium containing rapamycin, a mTOR inhibitor, were examined for cathepsin L expression at 5-dps. The presence of rapamycin lowered cathepsin L mRNA levels in control groups, but did not cause further decrease in FD cells. Data were collected from at least 5 independent experiments. CTL, control (cells without FD); FD, folate deficiency; CTSL, cathepsin L. Statistical data are shown in mean ± SEM. * *p*<0.05, **, *p* <0.01; ***, *p*<0.001, ****, *p*<0.0001

The impaired maturation of autolysosomes may result from various causes, including lysosomal dysfunction. Previously, we had shown that the expression of cathepsin L was altered in the cells and zebrafish displaying FD [18, 21]. Cathepsin L is a member of the cysteine proteinase family crucial to lysosomal activity and extracellular matrix (ECM) integrity. We found that the expression of cathepsin L in FD Huh7 cells were significantly decreased in a time-dependent manner (Fig. 7a and b). The apparent responsiveness of cathepsin L expression to folate status prompted us to examine cathepsin L promoter activity under FD condition. The genomic DNA encompassing the -2500 to 270 promoter region of zebrafish cathepsin L was cloned and transfected to Huh7 cells for promoter activity assay. A significantly decreased promoter activity was detected in the cells cultivated in FD medium for three days (Fig. 7c). These results support the folate responsiveness of cathepsin L expression and the contribution of decreased cathepsin L to FD-induced autophagic dysfunction. Adding Rapamycin, the inhibitor of phosphorylated mTOR, decreased cathepsin L expression in control cells, but did not further deteriorate the down-regulation of cathepsin L in FD cells (Fig. 7d). To investigate the expression of genes involved in autolysosome maturation, we conducted microarray analysis on FD embryos at 14 hpf, 30 hpf, and 120 hpf. However, no significant changes were observed in the expression levels of genes associated with autolysosome maturation within the expression profiles (Table. 1). These results suggest that FD and rapamycin work on the same pathway, that is FD inhibits cathepsin L expression via inhibiting mTOR phosphorylation.

### Folate supplementation alleviated the dysfunctional autophagic flux and lipid accumulation caused by cathepsin L inhibition

**Fig. 8:**
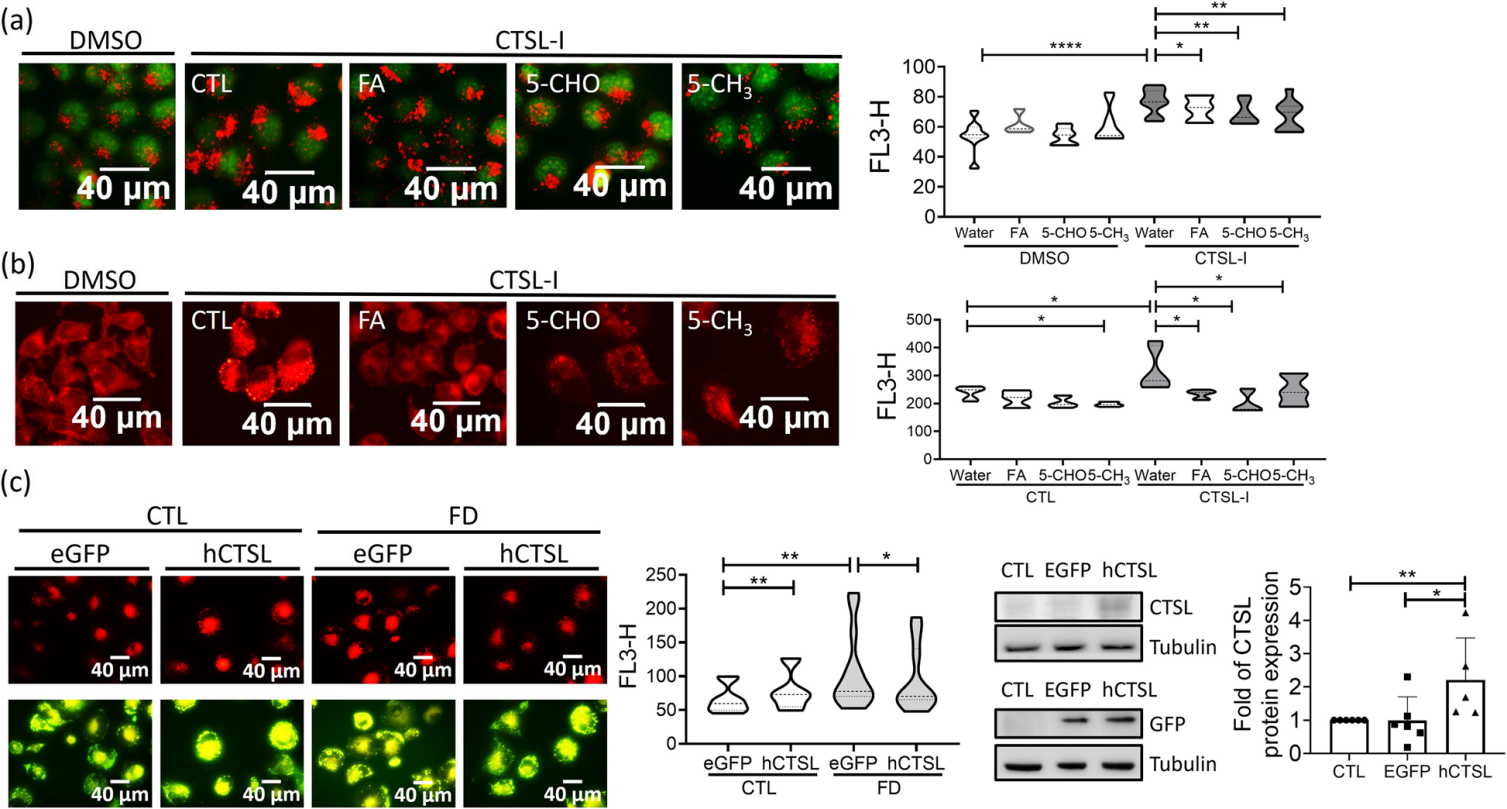
Inhibiting cathepsin L activity increased the number of acidic vesicles and lipid accumulation in Huh7 cells. **a**-**b** Huh7 cells grown in medium containing 40 µM cathepsin L inhibitor (CTSL-I) for 2 days were stained with acridine orange for acidic vesicles (**a**) and Nile red for lipid accumulation (**b**). Cells were imaged with fluorescence microscopy (left) and quantified for fluorescence intensity with flow cytometry (right). Increased numbers of red fluorescent puncta/intensity were found in FD cells, which were effectively prevented by folate supplementation. Data were collected from at least 6 independent experiments. **c** Cells transfected with hCTSL/eGFP/pCS2+ plasmids encoding recombinant human cathepsin L were subjected to Nile red staining for lipid deposition (left) and quantified with flow cytometry (middle). The expression of recombinant human cathepsin L was confirmed with Western blotting (right). Presented are the averaged results of at least five independent trials. CTL, control (cells without FD); FD, folate deficiency; CTSL, cathepsin L. Statistical data are shown in mean ± SEM. * *p*<0.05, **, *p* <0.01; ***, *p*<0.001, ****, *p*<0.0001. Scale bars = 40 µm

To confirm the causal link among lowered cathepsin L activity, disturbed autophagy and lipid accumulation, Huh7 cells were cultivated in regular medium containing cathepsin L inhibitor (CTSL-I) and stained with AO to assess autophagic flux. Examination with fluorescence microscopy and flow-cytometry revealed a significant increase in the number of acidic vesicle (i.e., autolysosome and lysosome) in the presence of cathepsin L inhibitor (Fig. 8a). This increase was prevented by simultaneously adding folic acid, 5-CHO-THF and 5-CH_3_-THF to medium. Flow cytometric analysis on the Nile red stained cells revealed apparent lipid accumulation in the cells exposed to cathepsin L inhibitor, which was also effectively prevented by folate supplementation (Fig. 8b). The FD-induced intracellular lipid accumulation was alleviated when cells were transfected with hCTSL/eGFP/pCS2+ plasmid expressing human cathepsin L (Fig. 8c). Together, these results show that folate supplementation effectively prevented the impaired autophagy and elevated lipid accumulation caused by lowering cathepsin L activity.

### Decreased cathepsin L expression and increased lipid accumulation also occurred to MTX-treated Huh7 cells

**Fig. 9:**
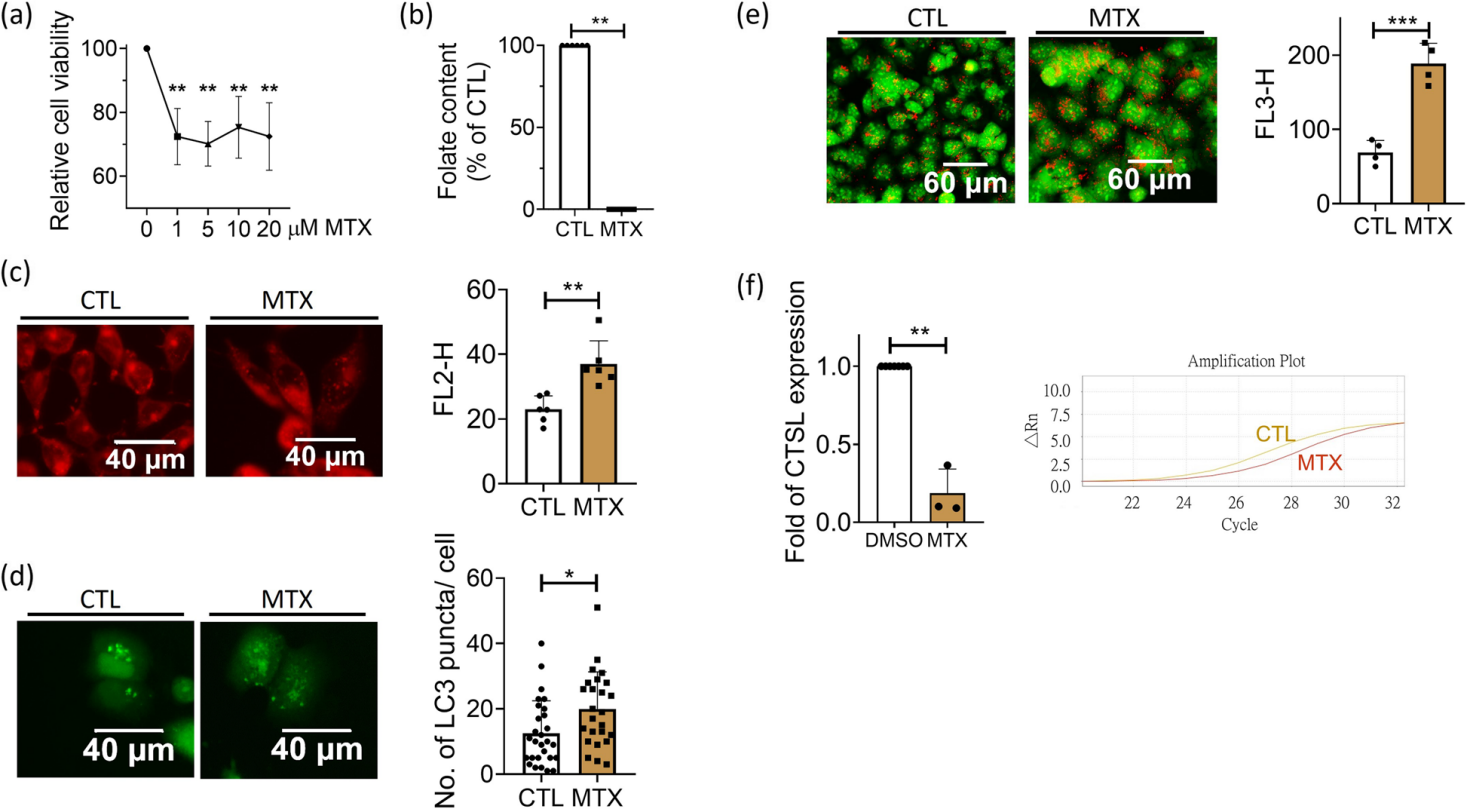
The folate deficiency caused by methotrexate altered autophagic activity and decreased cathepsin L expression in Huh7 cells. **a** Huh7 cells were examined for viability after cultivating in regular DMEM medium containing methotrexate (MTX) of indicated concentrations. An approximately 30% decrease in cell viability was observed in the presence of MTX at 1 µM and beyond. Data were collected from 5 independent experiments. **b** An almost complete depletion of intracellular folate content was reached after cells were cultivated in 5 µM MTX for 2 days. Data were collected from 6 independent experiments. **c**-**e** Cells grown in 5 µM MTX for 2 days were stained with Nile red for lipid deposition (**c**), transfected with plasmids encoding LC3-eGFP for characterizing autophagic activity (**d**) and subjected to Acridine orange staining for acidic vesicles (**e**). Increased lipid accumulation, autophagy and lysosomes/autolysosomes were apparent in MTX-treated cells. Data were collected from at least 3 independent experiments. **f** Cells were harvested 2 days after exposing to 5 µM MTX and examined for cathepsin L mRNA. A significant decrease in cathepsin L expression was found for MTX-treated cells. Presented are the averaged results of at least three independent trials. MTX, methotrexate. Statistical data are shown in mean ± SEM. * *p*<0.05, **, *p* <0.01; ***, *p*<0.001

The impact of disturbed cellular folate status on cathepsin L expressional regulation and lipid deposition was examined by cultivating Huh7 cells in the medium containing methotrexate (MTX), the anti-folate drug often used for the first-line chemotherapy and to disturb folate-mediated OCM in lab. Cell viability was decreased by approximately 30% in the presence of methotrexate at 1 µM concentration and beyond (Fig. 9a). An almost complete depletion of intracellular folate was reached when cells were grown in 5 µM methotrexate (Fig. 9b). Significantly increased lipid accumulation, LC3 puncta and AO staining positive signals, along with decreased cathepsin L transcript, were observed in methotrexate-treated cells (Fig. 9c-f). These results further support the interplay among disturbed intracellular folate status, autophagy, cathepsin L availability and lipid metabolism, echoing the responsiveness of cathepsin L expressional regulation and autophagic control to intracellular folate status.

### The down-regulation of hepatic cathepsin L expression and activation of autophagy in response to FD was also observed *in vivo*

**Fig. 10:**
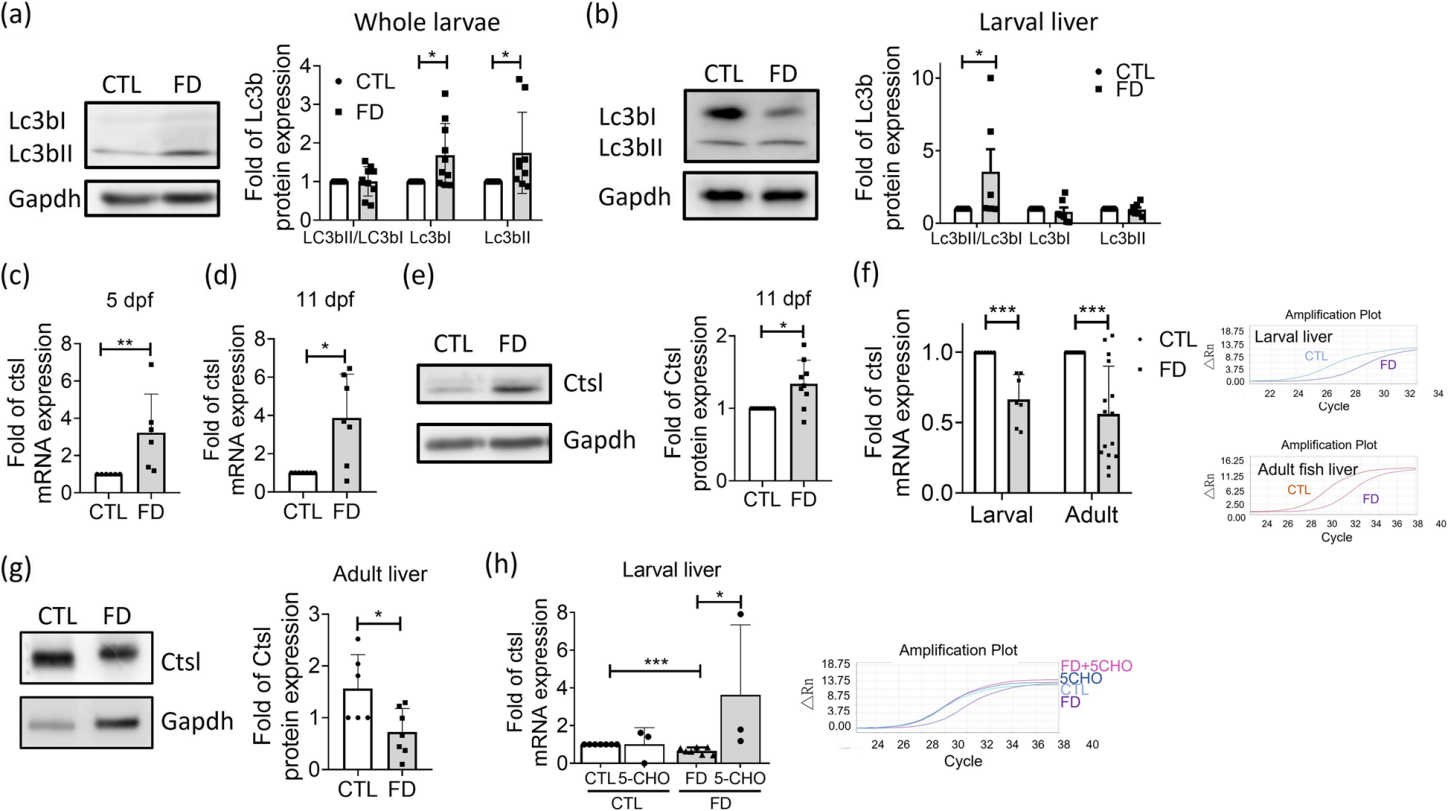
Up-/dysregulation of autophagy and down-regulation of cathepsin L expression also occurred in the liver of FD fish *in vivo*. **a**-**b** Cell lysates prepared from the whole larvae (**a**) and isolated liver from larvae of 11 dpf (**b**) were Western blotted for Lc3b and quantified with densitometry. Elevated Lc3bII/Lc3bI ratio was most apparent in FD larval liver, signifying an enhanced autophagosome formation, interrupted autophagosome-lysosome fusion, or lysosomal deregulation. Data were collected from at least 7 independent experiments. (**c**-**d**) Real-time PCR performed with the sample prepared from whole larvae of 5 dpf (**c**) and 11 dpf (**d**) revealed increased cathepsin L expression. Data were collected from at least 6 independent experiments. **e** Western blotting was conducted with the extract prepared from the whole larvae of 11 dpf for cathepsin L protein level, which revealed an increased expression for FD group. Data were collected from 9 independent experiments. **f**-**g** The liver-specific decrease in the expression of cathepsin L was observed both in the larvae of 11 dpf and adult fish of FD groups for both mRNA (**f**) and protein (**g**) levels. Data were collected from at least 6 independent experiments. **h** Larvae were induced for FD and supplementing with 1 mM 5-CHO-THF from 7 to 11 dpf before collected and examined for cathepsin L expression. Folate supplementation significantly increased the cathepsin L mRNA in FD larval liver. Presented are the averaged results of at least three independent trials with each of the sample prepared from 10-20 larvae. CTL, control (Tg-GGH/LR larvae without FD); FD, folate deficiency; 5-CHO, 5-formyl-THF. Statistical data are shown in mean ± SEM. * *p*<0.05, **, *p* <0.01; ***, *p*<0.001

Elevated Lc3b protein and the ratio of Lc3bII/ Lc3bI were found in the extracts of FD whole larvae and larval liver, supporting the notion of an activated initiation of autophagic pathway *in vivo* (Fig. 10a and b). The cathepsin L mRNA levels and protein in the FD whole larvae were increased (Fig. 10c-e), as previously described [21]. As expected, lowered cathepsin L transcripts and protein levels were found in FD samples when the fish livers were isolated from both larvae and adult fish and used for examination (Fig. 10f and g). In addition, supplementing with 5-CHO-THF successfully rescued the decreased expression of cathepsin L caused by FD (Fig. 10h). Together with the apparent lipid accumulation observed in FD larvae and adult fish, these results support that FD decreased cathepsin L expression and impaired autophagy, contributing to increased lipid deposition in hepatocytes.

## Discussion

**Fig. 11:**
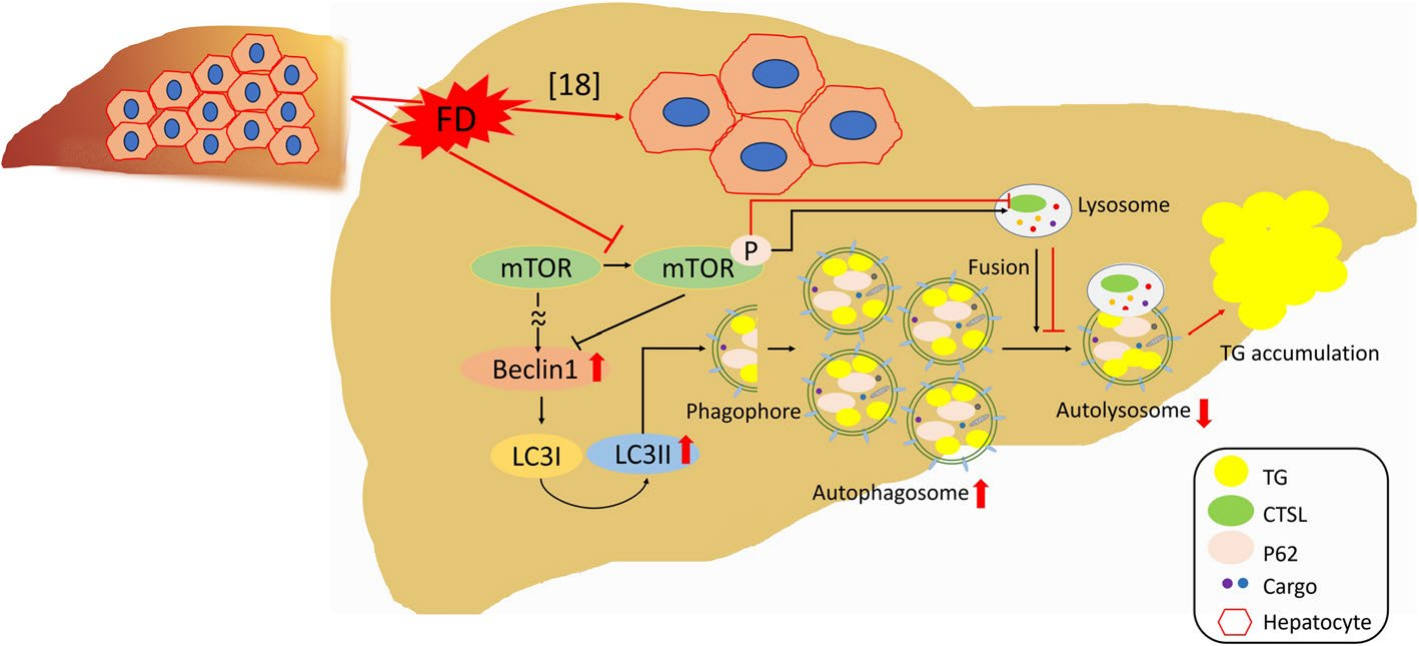
The prospective pathomechanism contributing to the FD-induced hepatic lipid accumulation. FD prevents mTOR phosphorylation, which enhances autophagic initiation. The decreased mTOR phosphorylation also down-regulates cathepsin L expression, which results in ineffective lysosomal activity and impaired autolysosome maturation, leading to disrupted autophagic flux and hepatic lipid accumulation

In our current studies, we have reported that FD activates autophagy but down-regulates the expression of cathepsin L. This leads to ineffective autophagic metabolism and lipid accumulation in the liver. Based on our results and findings from other researchers, we have proposed a prospective pathomechanism that explains how FD induces lipid accumulation in hepatocytes (Fig. 11). In this pathomechanism, FD reduces mTOR phosphorylation, which in turn activates autophagy. Decreased mTOR phosphorylation also results in the down-regulation of cathepsin L, a major lysosomal enzyme involved in lipid catabolism. This disruption impairs autolysosomal maturation, obstructs autophagic flux, and increases the autophagosome/autolysosome ratio, ultimately leading to lipid accumulation in liver cells. Additionally, we found similar impeded cathepsin L expression and autophagic activity in both FD cells and liver cells treated with methotrexate. This provides a pathomechanism for the methotrexate-induced fatty liver and hepatoxicity observed in patients undergoing methotrexate chemotherapy [22]. We also found that folate supplementation can restore impaired autophagy and prevent lipid accumulation caused by the inhibited activity of cathepsin L, regardless of the presence or absence of FD. This finding offers a potential strategy for treating lysosomal/autophagic dysfunction-associated hepatopathology, including non-FD-induced fatty liver.

Our data reveal a tissue-specificity for the cellular responsiveness to FD, specifically for the expressional regulation of cathepsin L. Initially, we were puzzled by the inconsistence between hepatic lipid accumulation and increased cathepsin L expression in FD whole larvae. This is because we had previously shown that FD increased the expression of cathepsin L when the whole larvae were used for analysis [21]. Of all the lysosomal proteases, cathepsins L is one of the most abundant enzymes with their lysosomal concentrations equivalent to 1 mM [23]. One would expect that higher cathepsin L expression would increase autolysosomal activity and assist autophagy-mediated lipid catabolism [24]. The decrease of cathepsin L expression specifically in fish liver and hepatocytes via modulating mTOR signaling offers an explanation for these seemingly conflicting observations. It is intriguingly to note that significant increase in the expression of proton-couple folate transporter (PCFT) was observed in FD Huh7 cells (Fig. S2). PCFT is a member of folate transporting system mediating the intestinal absorption of folate. PCFT has been reported to assist in the folate sensing activity of mTOR by functioning as a mechanistic link between folate availability and mTOR signaling in trophoblast cells [25]. Further investigation is warranted to determine whether the PCFT-mediated modulation of mTOR signaling also plays a role in the tissue-specificity of FD-induced mTOR-mediated autophagic control.

The contribution of cathepsin enzymes to the tissue-specificity of FD-induced autophagic dysfunction has been reported before and further addressed in the current study. Previously, we demonstrated that FD disrupts the autophagic-lysosomal pathway in neuroblastoma cells and fish brain by down-regulating cathepsin B expression, which led to Alzheimer's-like pathology [18]. Cathepsin B is one other major cysteine protease in the family of cathepsins constituting lysosomal enzymes. Despite the significant decrease found in blastoma cells and fish brain in response to FD, the expression of cathepsin B was not significantly affected in both FD Huh7 cells and larvae (Fig. S3). Our findings echo the reports that FD also induced autophagic dysfunction in tissues other than liver, including cortex and placenta [26, 27]. These results further emphasize the tissue-specific manner of the expressional regulation for cathepsin enzymes, as well as the close interplay among cellular folate status, cathepsin expression, and lysosomal-autophagic control.

We found that supplementing with folate can restore compromised autophagy caused by the suppression of cathepsin L activity, regardless of whether it is associated with disturbed folate-mediated OCM, offering a potential treatment for illnesses linked to autophagic dysfunction. These results are encouraging since impaired cathepsin L activity, either due to altered expression or the presence of inhibitor, has been implied in many diseases, including lysosomal storage disorders, osteoporosis, cancer, neurodegenerative disorders, and cardiovascular diseases [28–31]. Our findings suggest that individuals with the aforementioned illnesses, even in the absence of apparent FD symptoms, may still benefit from folate supplementation. On top of a host of benefits for improving general health and preventing/managing diseases, folate supplementation has been considered a practical and accessible option for various populations with the advantages of easy accessibility, cost-effectiveness, safety and minimal side effects. We are convinced that comprehending the tissue-specific impact brought by FD to cellular pathways and metabolism, including cathepsin-related autophagic control, shall be rewarding for disease manifestation and pave the way to developing the strategies tailoring therapeutic interventions to target specific tissues or organs.

Significant alterations in several features of mitochondria, including perinuclear localization, elevated fusion-fission dynamics, and altered oxygen consumption rate and mitochondrial membrane potential have been found in FD Huh7 cells. The hepatic mitochondrial one-carbon metabolism is uniquely important because the liver, acting as a folate reservoir, contains the most abundant folate enzymes and actively mediates one-carbon metabolism among organs [32]. Moreover, serine, one of the major one-carbon sources, is primarily catabolized to provide one-carbon unit through the mitochondrial one-carbon metabolism pathway [33, 34]. The intracellular localization of mitochondria and their distribution are highly regulated processes since proper mitochondrial positioning is essential for maintaining cellular homeostasis and energy balance. The redistribution of mitochondria observed in FD Huh7 cells signifies a cellular response to the stress conditions caused by FD and the ultimate lipid accumulation, which may convey an adaptive response critical for cell survival and homeostasis. We have reported previously that disturbing folate-mediated OCM homeostasis impeded F-actin polymerization both in hepatocytes and in developing embryos [19, 35]. F-actin is crucial to mitochondria distribution and dynamics through its involvement in the anchoring, positioning and transport of mitochondria [36, 37]. Therefore, we could not exclude the possibility that the interfered cytoskeletal dynamics also contribute to the mitochondrial relocation and even the autophagic dysfunction observed in FD liver cells.

One other intriguing finding is that the results of JC-1 staining seem to suggest a healthier and more active mitochondria in FD cells. JC-1 is a cationic dye that selectively accumulates in the mitochondria and undergoes a reversible shift in emission color depending on the mitochondrial membrane potential. In general, JC-1 forms red-fluorescent J-aggregates in healthy, polarized mitochondria with high membrane potential; while in depolarized mitochondria with low membrane potential, it emits green fluorescence as a monomeric form. It should be noted that the staining efficacy of JC-1 is influenced by the surface-to-volume (S/V) ratios of the cell [38]. We have previously reported that FD induces hepatic cell size enlargement [19]. It is likely that the elevated JC-1 aggregates/monomers are a result of the increased surface-to-volume (S/V) ratios of FD cells. It should be noted that mitochondrial DNA is particularly vulnerable to oxidative damage because of its close proximity to reactive oxygen species (ROS) generated during oxidative phosphorylation. In addition, mitochondria have their own pool of nucleotides to support the synthesis of mitochondria DNA and RNA. Without sufficient one-carbon units, the mitochondrial DNA replication and repair may be impaired, leading to mitochondrial dysfunction and compromising the overall energy production capacity of the cell. Therefore, FD may have a deteriorating impact on mitochondrial biology due to the antioxidant activity of folate and its crucial role in nucleotide formation. The question of whether mitophagy, a process involving the autophagic degradation of mitochondria, has taken place and contributed to FD-induced autophagic dysfunction and lipid accumulation, warrants further study.

## Conclusions

Disturbed folate-mediated OCM, either due to folate deficiency or the presence of anti-folate drug, decreases mTOR phosphorylation and down-regulates cathepsin L expression, leading to compromised lysosomal function, impaired autolysosome maturation and enhanced mitophagy. Moreover, folate supplementation improves autophagic impairment and alleviates lipid accumulation caused by the inhibited activity of cathepsin L. These findings offer a promising approach for treating hepatopathology related to lysosomal/autophagic dysfunction, including non-folate deficiency-induced fatty liver.

## Methods

### Material

5-methyl-THF was gifts from Dr. Moser (Merck Eprova AG, Switzerland). 5-formyltetrahydrofolate was purchased from Schircks Laboratories (Bauma, Switzerland). Fetal bovine serum (FBS), trypsin-EDTA, and alamarBlue cell viability reagent were purchased from Invitrogen, Thermo Fisher Scientific Inc. (CA, USA). Total Cholesterol and Cholesteryl Ester Colorimetric/Fluorometric Assay Kit and Triglyceride Quantification Colorimetric/Fluorometric Kit were purchased from BioVision, Inc. (SF, USA). Cathepsin L Inhibitor was purchased from Cayman Chemical (MI, USA). AST assay and ALT assay were purchased from Roche (Basel, CH). Dual-Luciferase® Reporter Assay was purchased from Promega (Madison, Wisconsin, USA). Mouse anti-P62 antibodies was purchased from Santa Cruz (CA, USA). Rabbit anti-GAPDH antibody was purchased from GeneTex (CA, USA). Rabbit anti-LC3b antibody was purchased from Abcam plc. (Cambridge, UK). The plasmid encoding GFP-LC3 and tandem fluorescent-tagged LC3 (tfLC3) were gifts from Dr. Tamotsu Yoshimori and Dr. Noboru Mizushima/University of Tokyo [20, 39]. All other chemicals, including folic acid (FA), methotrexate (MTX), Oil Red O (ORO), acridine orange, and proteinase inhibitor cocktail were purchased from Sigma-Aldrich Chemical Co. (WI, USA).

### Fish (*Danio rerio*) lines and maintenance

Zebrafish, including wild-type AB strain, was purchased from NTHU-NHRI Zebrafish Core Facility (supported by MOST 104-2321-B-001-045), Taiwan and bred and maintained at 28℃ in a 14-hour light/10-hour dark diurnal cycle following the standard procedure [40]. The folate deficiency zebrafish transgenic line Tg(*lfabp*:mCherry/*hsp70*:eGFP-γGH) were previously established in our lab [18] and regularly maintained at 25℃. All usage and experiments of adult and embryo were approved by the Institutional Animal Care and Use Committee, National Cheng Kung University, Tainan, Taiwan (IACUC Approval No. 106086 and No.109172).

### Induction of folate deficiency

Tg(*lfabp*:mCherry/*hsp70*:eGFP-γGH) larvae were heat-shocked at 5,6, and 7 dpf at 37.5℃ for an hour each time. Tg(*lfabp*:mCherry/*hsp70*:eGFP-γGH) adult fish were heat-shocked six times (38.2 °C for 6 hour each time) with a 2-day interval between each induction.

### Compounds treatment

Most of the compounds and inhibitors were prepared as stocks in water or DMSO, added to embryo-containing E3 buffer and refreshed every other day unless otherwise mentioned. The final concentrations used were: FA 1mM, 5-formyltetrahydrofolate (5-CHO-THF) 1 mM, 5-methyltetrahydrofolate (5-CH_3_-THF), and cathepsin L inhibitor 40 µM.

### Quantification for gene expression

The expression of gene was examined with real-time PCR and Western blotting as previously described [41]. The primers used were: 5’- GGCAGGATGATGTTTGCTTT-3’ (forward) and 5’- AGAATGCAGAGAAGGCTCCA-3’ (reverse) for zebrafish cathepsin Lb (ctsl1b); 5’- GTTGCTATTGATGCAGGTCATGA -3’ (forward) and 5’- ACTGCTACAGTCTGGCTCAAAATAAA -3’ (reverse) for human cathepsin L (CTSL); 5’-GTCCACCGCAAATGCTTC-3’ (forward) and 5’-ATTGCCGTCACCTTCACC-3’ (reverse) for zebrafish and human β-actin as internal control.

### Cell culture

Huh7 cells were regularly maintained in Dulbecco’s Modified Eagle Medium (DMEM, Gibco, 12800-017) containing 10% FBS in 5% CO_2_ at 37℃. For inducing folate deficiency, cells were cultured in special Minimum Essential Medium-α without folic acid (Gibco, 97-5104EL) or specific Dulbecco’s Modified Eagle Medium (HIMEDIA, AT006F) in the presence of 5% charcoal-treated FBS. For the control group, additional folic acid was added to medium to reach the final concentration of 0.004 g/L.

### Oil Red O staining

Zebrafish larvae were fixed in 4% paraformaldehyde (PFA)/ phosphate buffered saline (PBS) at 4℃ and subsequently washed three times with PBS. The larvae were then sequentially pre-incubated in 20%, 40%, 60%, and 100% propylene glycol for 20 minutes each at room temperature. To stain the larvae, a fresh 0.5% ORO solution in 100% propylene glycol was applied at room temperature overnight. After staining, the larvae were subjected to a 20-minute fading step with 100% propylene glycol at room temperature and then stored in 80% glycerol. For frozen sections, they were pre-incubated in 100% and 85% propylene glycol for 5 minutes each at room temperature. Subsequently, the sections were stained with a freshly prepared 0.5% ORO solution at room temperature overnight. After staining, the sections were washed with ddH_2_O and nuclear staining was performed using Mayer's hematoxylin for 30 seconds. Finally, the sections were washed with ddH_2_O and mounted with Marinol.

### Cholesterol assay

The cholesterol content of Zebrafish larvae, Huh7 cells, and the culture medium was assessed using the Cholesterol-Cholesteryl Ester Quantification kit. Zebrafish larvae and Huh7 cells were extracted in the solvent containing chloroform: isopropanol: NP-40 (7:11:0.1). The supernatant was collected and air dried at 50℃. The resulting pellet was dissolved in assay buffer and mixed with cholesterol assay buffer, cholesterol probe, cholesterol enzyme mix, and cholesterol esterase. The samples were incubated for 1 hour at 37℃. The fluorescent signal was then measured at Ex/Em= 535/587 nm using a FlexStation 3.

### Triglyceride assay

Triglyceride content of zebrafish larvae, Huh7 cells, and culture medium were measured using PicoProbe Triglyceride Fluorometric assay kit following the manufacture’s instruction. Zebrafish larvae and Huh7 cells were collected and sonicated in ice cold TG assay buffer. The supernatant was collected by centrifugation. Lipase and sample were added to 96-well a plate and incubated at 37℃ for 20 min. Reaction mix were added to each well and incubated at 37℃ for 20 min in the dark. The fluorescent signal was examined at Ex/ Em= 535/ 587 nm with FlexStation 3.

### Nile red staining

Huh7 cells were stained with a 300 nM Nile red solution in PBS at room temperature for 15 minutes in the dark. After staining, the cells were washed with PBS and then harvested. The stained cells were prepared at a concentration of 1*10^6^ cells/ml and were subjected to flow cytometry analysis using a FACS CALIBUR (BD) instrument, following the manufacturer's instructions for neutral lipid determination.

## Aspartate aminotransferase (AST) and alanine aminotransferase (ALT) assay

AST and ALT levels in both cell lysate and the used culture medium were assessed using an AST and ALT kit. To prepare the cell lysates, cells were collected and lysed in RIPA buffer, followed by sonication on ice. After centrifugation, the supernatant was collected and incubated with R1 reagent at 37℃ for 5 minutes before adding R3 reagent. The sample were monitored for absorbance at 340 nm at 37℃ for 5 minutes.

### Cell viability

Cell viability of Huh7 cells were assessed using AlamarBlue following the manufacture’s instruction. Huh7 cells were grown in CTL or FD medium with/ without folate supplementation. At day 8, 10% (v/v) alamarBlue reagent in CTL or FD medium were added to 96-well plates and incubated at 37℃ for 2 hr in the dark. The supernatant was collected and transferred to a new 96-well plates and examined for the absorbance at 570 and 600 nm.

### Transfection

Huh7 cells were seeded in 24-well plates and cultured in CTL or FD medium. Cells were transfected with plasmid encoding GFP-LC3, tandem fluorescent-tagged LC3 (tfLC3) or hCTSL/EGFP/pCS2+ with Lipofectamine 3000 reagent following the manufacturer’s protocol (Invitrogen L3000015) at 7-day post seeding (dps). Cells were observed for puncta formation under fluorescence microscope at 8 dps.

### Acridine orange

Huh7 cells were seeded in 24-well plates and stained with 5 µg/ml acridine orange at 37℃ for 30 min in the dark before examined with fluorescence microscopy.

### Measurement of oxygen consumption

Huh7 cells were cultured in CTL or FD medium for 8 to 9 days and examined for oxygen consumption using the Oxygraph-2k (O2k, OROBOROS INSTRUMENTS, Austria). Cells (2.1* 10^6^ cells/ 2.1 ml culture medium) were added to the calibrated O2k chambers. After a 10-minute waiting period to record routine respiration, 0.525 mM oligomycin was added to inhibit ATP synthase for measuring proton leak respiration. FCCP (1 µl of 1.155 mM solution/injection) was repeatedly introduced into the O2k chamber sequentially until maximum respiration achieved. Finally, 5.25 M sodium azide was added to inhibit cytochrome c oxidase for measuring non-mitochondrial respiration.

### JC-1 staining

Huh7 cells were incubated with 10 µg/ml JC-1 (5,5',6,6'-tetrachloro-1,1',3,3'-tetraethylbenzimidazolylcarbocyanine iodide) at 37℃ for 15 minutes in the dark and then washed with PBS. After examination with fluorescence microscopy, the cells were harvested by trypsinization and subjected to flow cytometry analysis using a FACS CALIBUR (BD) instrument.

### Cloning for human CTSL

The human cathepsin L (CTSL) coding sequences was PCR amplified from Huh7 cDNA library with primers (forward: 5’- ATCGA TATGA ATCCT ACACT CATCC TTGC-3’; reverse: 5’- CCTCA GGTCA CACAG TGGGG TAGCT GG-3’) designed based on the coding sequence available in the Nucleotide database in NCBI (NM_ 001257971.2). The PCR products were cloned into the pGEM^®^-T Easy vector. This human CTSL coding sequence was also sub-cloned to pCS2+ vector via Bsu36I and ClaI restriction sites for expression.

### Promoter assay

The promoter activity assay was performed using dual-luciferase reporter assay following the manufacturer’s protocol (Promega E1910) and the protocol described previously [42]. For sample preparation, Huh7 cells were cultured in CTL or FD medium and transfected with plasmid constructs encompassing Ctsl1b promoter -2500 to 270 regions cloned to pGL3-Basic at 1-day post seeding. Cells were harvested 2 days after transfection and analyzed for promoter activity.

### Statistical analysis and reproducibility

The statistical significance was calculated with Student's t-tests and Mann-Whitney nonparametric U test at 95% confidence intervals using the software GraphPad Prism 8 (GraphPad Software; San Diego, CA) with the error bars representing s.e.m.

### Study approval

All usage and experiments of adult and embryo were approved by the Institutional Animal Care and Use Committee, National Cheng Kung University, Tainan, Taiwan (IACUC Approval No. 106086 and No. 109172).

### Supplementary Information

Additional file 1: Fig. S1. The expression of genes related to lipid metabolism in FD fish and Huh7 cells. No significant and consistent alteration in the expression of the genes examined was identified among FD Huh7 cells and the liver of larvae and adult fish.

Additional file 2: Fig. S2. The expression of PCFT in FD Huh7 cells was increased.

Additional file 3: Fig. S3. The expression of CTSB in larvae and Huh7 cells was not affected by FD.

Additional file 4: Fig. S4-S15. Images of the full immunoblots.

Additional file 5: Table 1. The changes in expression levels of genes related to autolysosome maturation, as determined by microarray data.

Additional file 6. The individual data values for Fig. 2a, 2e, 2h, 2i, 3a, 3b, 3d, 3e, 4f, 6b, 7a, 7d, 8a, 8c, 9a, 9e, 9f, 10h, S1b, S2, S3a, S3c.

## Data Availability

All data generated or analysed during this study are included in this published article and its supplementary information files. The individual data values of this paper are included in an additional Excel file.

## Abbreviations

5-CH_3_-THF: 5-methyltetrahydrofolate
5-CHO-THF: 5-formyltetrahydrofolate
ALT: Alanine aminotransferase
AST: Aspartate aminotransferase
CTSL: Cathepsin L
CTSL-I: Cathepsin L inhibitor
DMEM: Dulbecco’s Modified Eagle Medium
dps: Day post seeding; ECM: Extracellular matrix
FA: Folic acid
FBS: Fetal bovine serum
FD: Folate deficiency
Tg: (lfabpmCherry/hsp:eGFP-γGH)
HCC: Hepatocellular carcinoma
MAFLD: Metabolic-associated fatty liver disease
mTOR: Mechanistic target of rapamycin
MTX: Methotrexate
NASH: Nonalcoholic steatohepatitis
OCM: One-carbon metabolism
ORO: Oil Red O
PBS: Phosphate buffered saline
PCFT: Proton-couple folate transporter
PFA: Paraformaldehyde
tfLC3: Tandem fluorescent-tagged LC3
