# Supplementary material for: Disturbed intracellular folate homeostasis impairs autophagic flux and increases hepatocytic lipid accumulation
Source: BMC Biol. 2024 Jul 2;22:146. doi: 10.1186/s12915-024-01946-6 (PMC11220954; doi:10.1186/s12915-024-01946-6)
Supplement: Supplementary file 1 — Additional file 1: Fig. S1. The expression of genes related to lipid metabolism in FD fish and Huh7 cells. No significant and consistent alteration in the expression of the genes examined was identified among FD Huh7 cells and the liver of larvae and adult fish. [file 12915_2024_1946_MOESM1_ESM.docx]

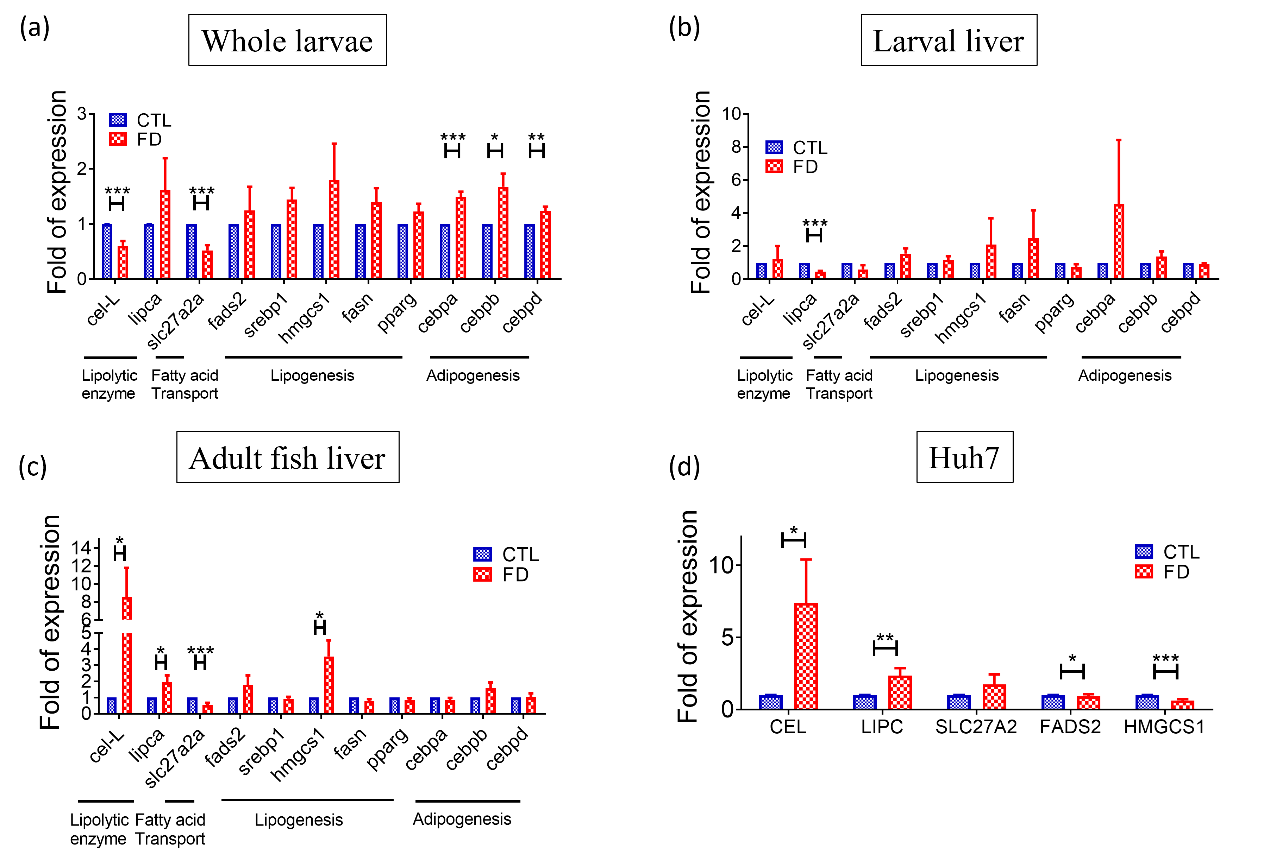


**Figure S1. The expression of genes related to lipid metabolism in FD fish and Huh7 cells.** The expression of genes participating in lipolysis, fatty acid transport, lipogenesis, and adipogenesis in whole larvae (a), liver isolated from larvae at 11 dpf (b) and adult fish (c), and Huh7 (d) were characterized with real-time PCR. No significant and consistent alteration in the expression of the genes examined was identified among FD Huh7 cells and the liver of larvae and adult fish. *Cel-L*, carboxyl ester lipase-like; *lipca*, hepatic lipase; *slc27a2a*, fatty acid transporter; *fads2*, fatty acid desaturase; *srebp1*, sterol regulatory element binding transcription factor 1; *hmgcs1*, HMG CoA synthase; *fasn*, fatty acid synthase; *pparg*, peroxisome proliferator-activated receptor gamma; *cebpa*, CCAAT enhancer binding protein alpha; *cebpb*, CCAAT enhancer binding protein beta; *cebpd*, CCAAT enhancer binding protein delta; CTL, control (cells or larvae without FD); FD, folate deficiency. Presented are the averaged results of at least three independent trials. Statistical data are shown in mean ± SEM. * p<0.05, **, p <0.01; ***, p<0.001.
