# Supplementary material for: Disturbed intracellular folate homeostasis impairs autophagic flux and increases hepatocytic lipid accumulation
Source: BMC Biol. 2024 Jul 2;22:146. doi: 10.1186/s12915-024-01946-6 (PMC11220954; doi:10.1186/s12915-024-01946-6)
Supplement: Supplementary file 2 — Additional file 2: Fig. S2. The expression of PCFT in FD Huh7 cells was increased. [file 12915_2024_1946_MOESM2_ESM.docx]

**
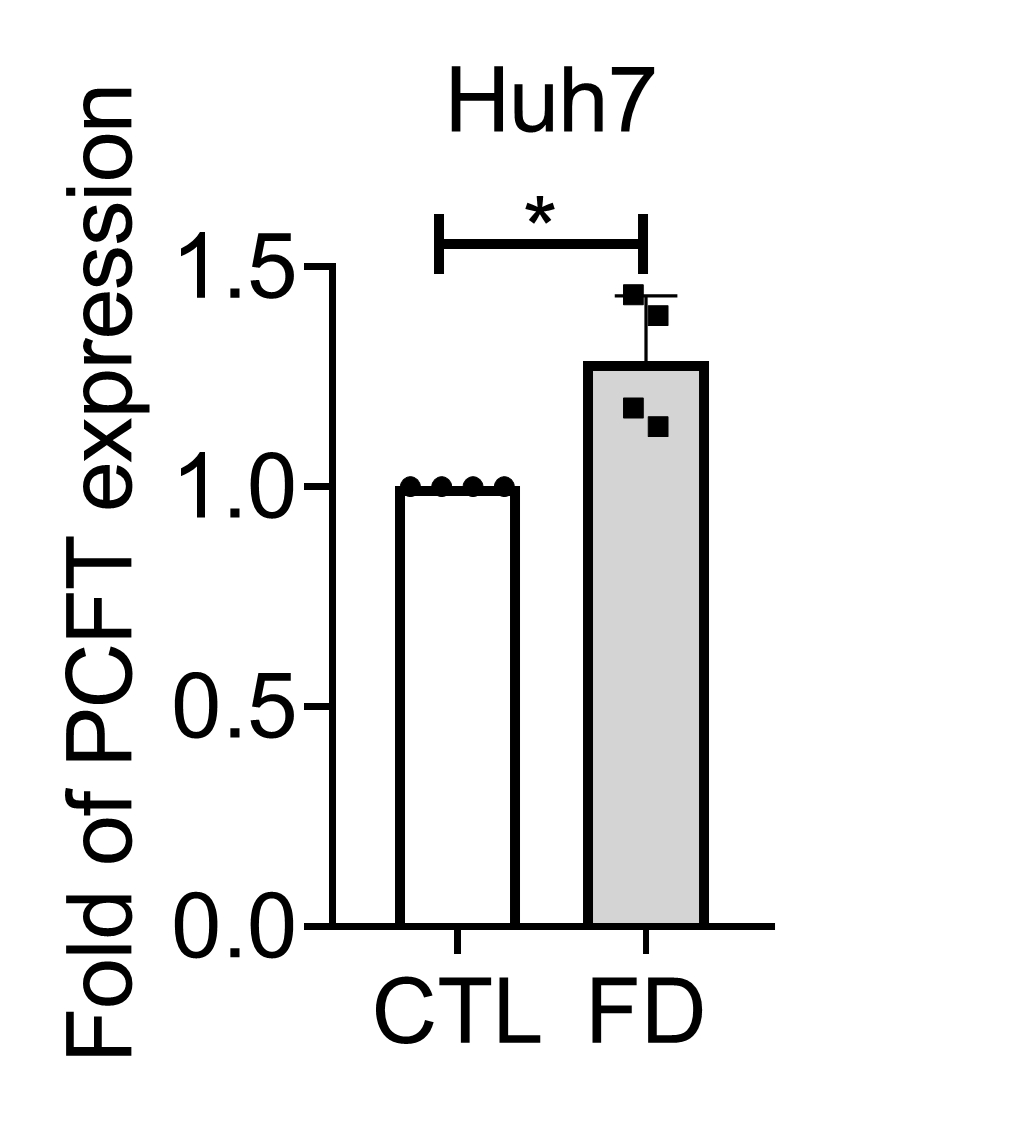
**

**Figure S2. The expression of PCFT in FD Huh7 cells was increased.** The mRNA levels of PCFT in Huh7 cells cultivated in control and FD medium were examined with RT-PCR. PCFT, proton-coupled folate transporter; CTL, control (cells without FD); FD, folate deficiency. Presented are the averages of at least three independent trials. Statistical data are shown in mean ± SEM. * p<0.05, **, p <0.01; ***, p<0.001.
