# Supplementary material for: Disturbed intracellular folate homeostasis impairs autophagic flux and increases hepatocytic lipid accumulation
Source: BMC Biol. 2024 Jul 2;22:146. doi: 10.1186/s12915-024-01946-6 (PMC11220954; doi:10.1186/s12915-024-01946-6)
Supplement: Supplementary file 3 — Additional file 3: Fig. S3. The expression of CTSB in larvae and Huh7 cells was not affected by FD. [file 12915_2024_1946_MOESM3_ESM.docx]

**
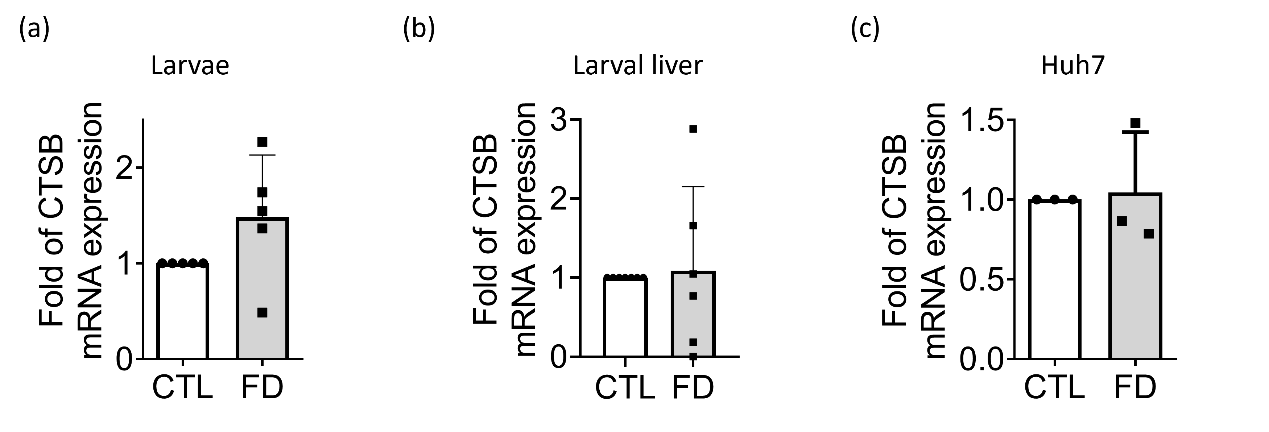
**

**Figure S3. The expression of CTSB in larvae and Huh7 cells was not affected by FD.** The mRNA levels of CTSB in whole larvae (a), liver isolated from larvae at 11 dpf (b), and Huh7 (c) were characterized with real-time PCR. CTSB, cathepsin B; CTL, control (cells or larvae without FD); FD, folate deficiency. Presented are the averaged results of at least three independent trials. Statistical data are shown in mean ± SEM. * p<0.05, **, p <0.01; ***, p<0.001.
