# Supplementary material for: Disturbed intracellular folate homeostasis impairs autophagic flux and increases hepatocytic lipid accumulation
Source: BMC Biol. 2024 Jul 2;22:146. doi: 10.1186/s12915-024-01946-6 (PMC11220954; doi:10.1186/s12915-024-01946-6)
Supplement: Supplementary file 4 — Additional file 4: Fig. S4-S15. Images of the full immunoblots. [file 12915_2024_1946_MOESM4_ESM.docx]

**Figure S4**

**
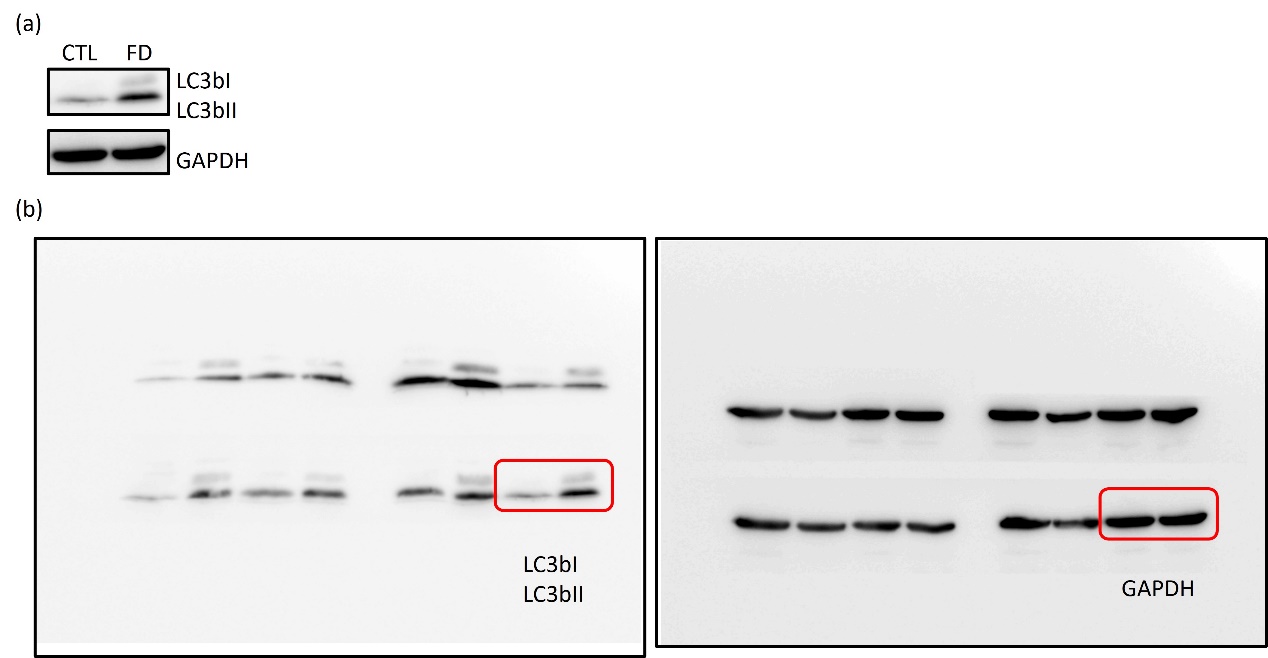
**

**Figure S4. The original Western blotting results for LC3b/GAPDH analysis.** (A) The original cropped Western blotting results as shown in Figure 4a in the manuscript. (B) The original full-length Western blots for the analyses on LC3b and GAPDH.

**Figure S5**

**
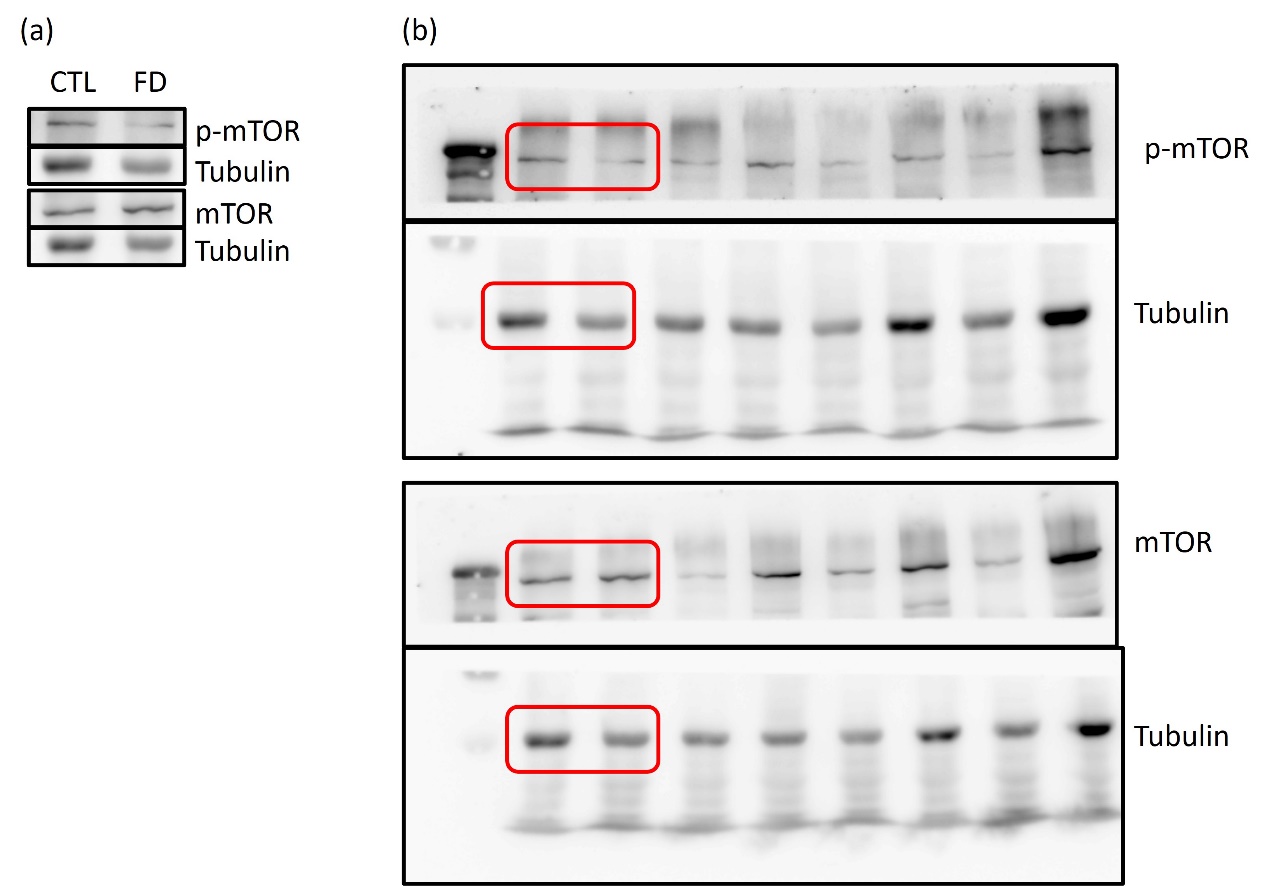
**

**Figure S5. The original Western blotting results for mTOR/Tubulin analysis.** (A) The original cropped Western blotting results as shown in Figure 4c in the manuscript. (B) The original full-length Western blots for the analyses on mTOR and Tubulin.

**Figure S6**

**
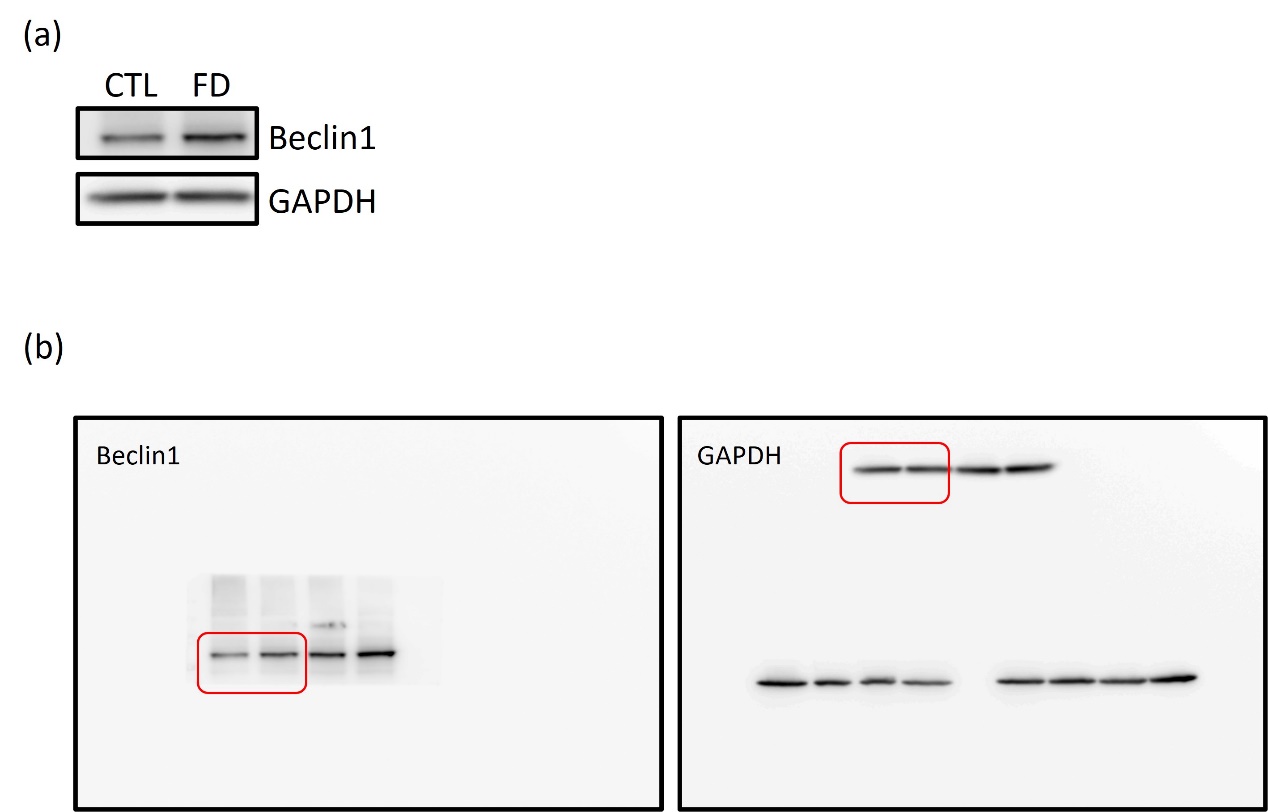
**

**Figure S6. The original Western blotting results for Beclin1/GAPDH analysis.** (A) The original cropped Western blotting results as shown in Figure 4d in the manuscript. (B) The original full-length Western blots for the analyses on Beclin1 and GAPDH.

**Figure S7**

**
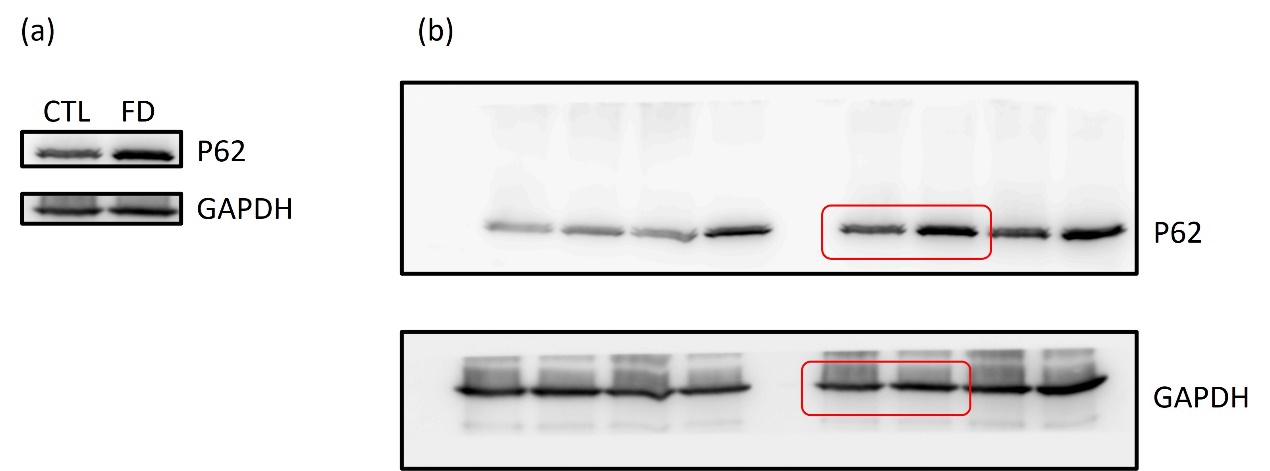
**

**Figure S7. The original Western blotting results for P62/GAPDH analysis.** (A) The original cropped Western blotting results as shown in Figure 4f in the manuscript. (B) The original full-length Western blots for the analyses on P62 and GAPDH.

**Figure S8**

**
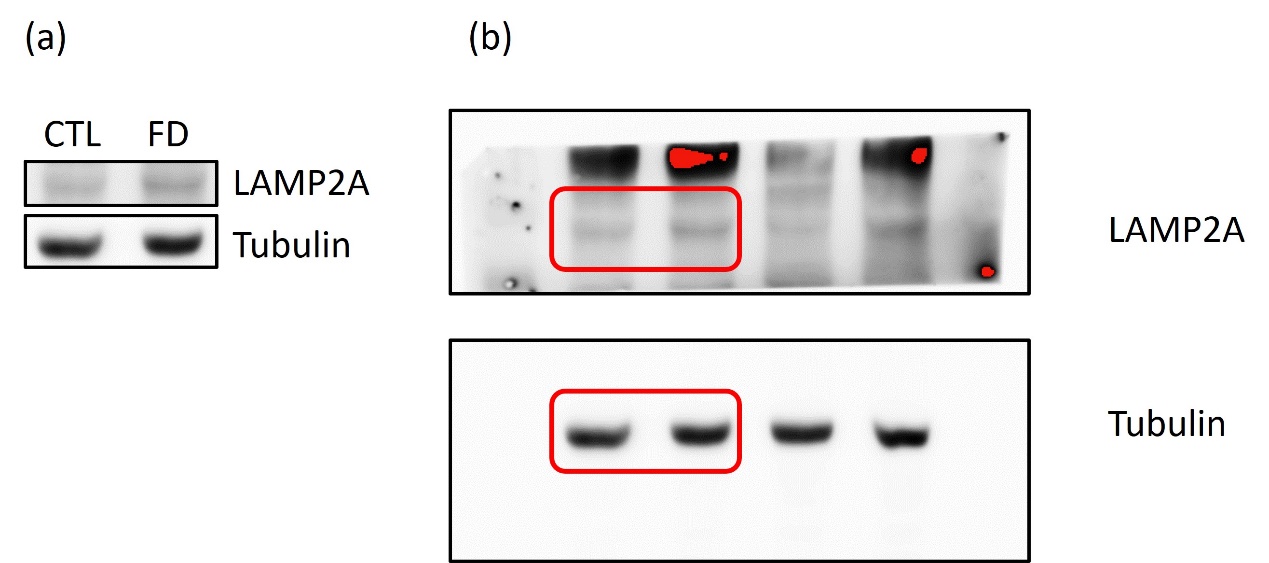
**

**Figure S8. The original Western blotting results for LAMP2/Tubulin analysis.** (A) The original cropped Western blotting results as shown in Figure 5c in the manuscript. (B) The original full-length Western blots for the analyses on LAMP2 and Tubulin.

**Figure S9**

**
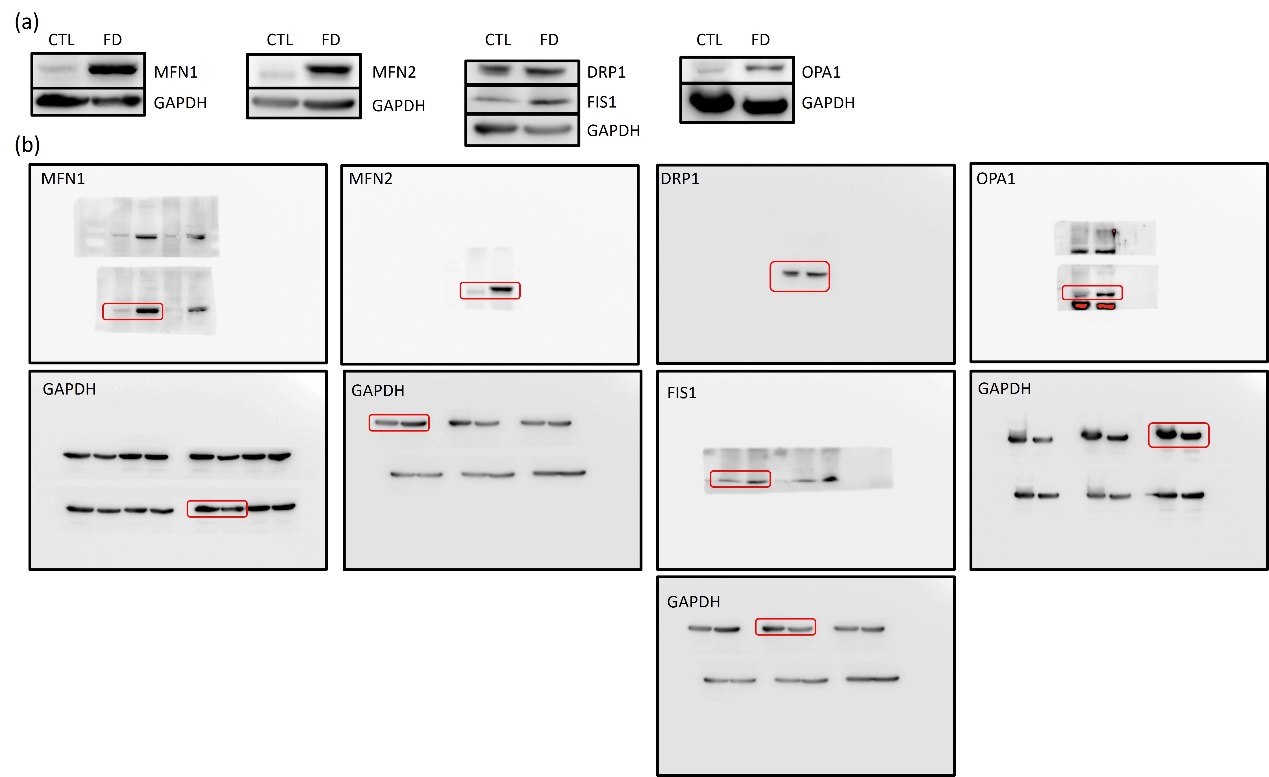
**

**Figure S9. The original Western blotting results for MFN1/GAPDH, MFN2/GAPDH, DRP1/GAPDH, FIS1/GAPDH, and OPA1/GAPDH analysis.** (A) The original cropped Western blotting results as shown in Figure 6b in the manuscript. (B) The original full-length Western blots for the analyses on MFN1, MFN2, DRP1, FIS1, OPA1, and GAPDH.

**Figure S10**

**
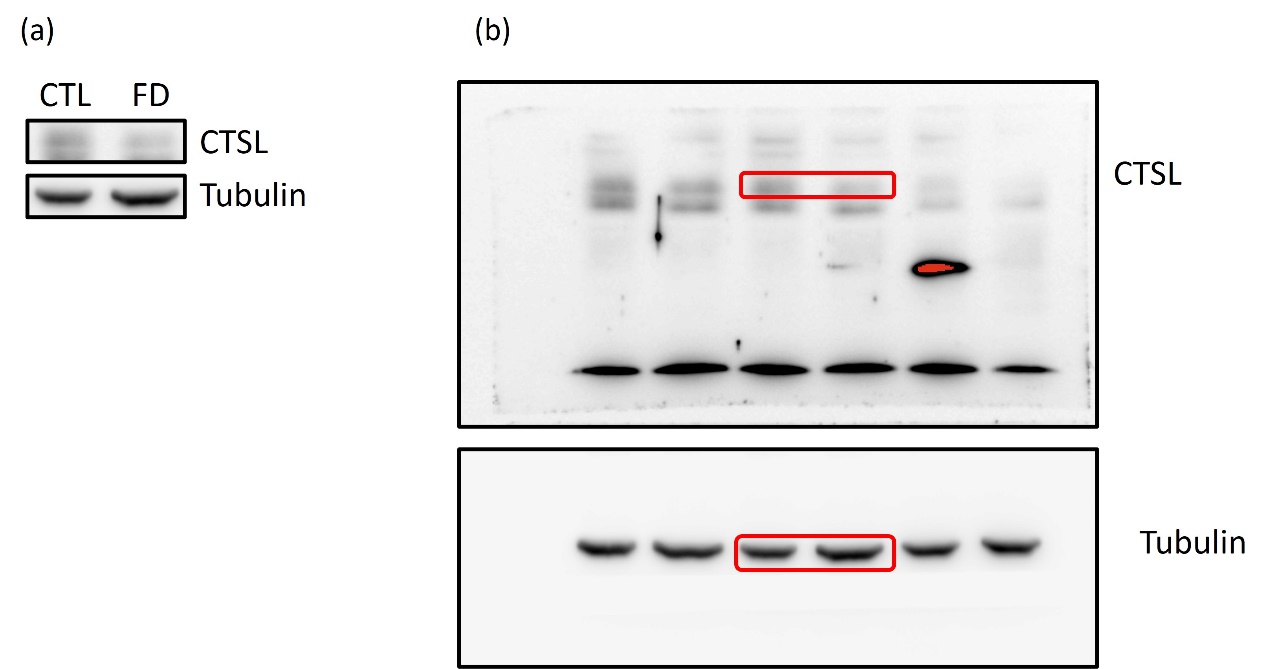
**

**Figure S10. The original Western blotting results for CTSL/Tubulin analysis.** (A) The original cropped Western blotting results as shown in Figure 7b in the manuscript. (B) The original full-length Western blots for the analyses on CTSL and Tubulin.

**Figure S11**

**
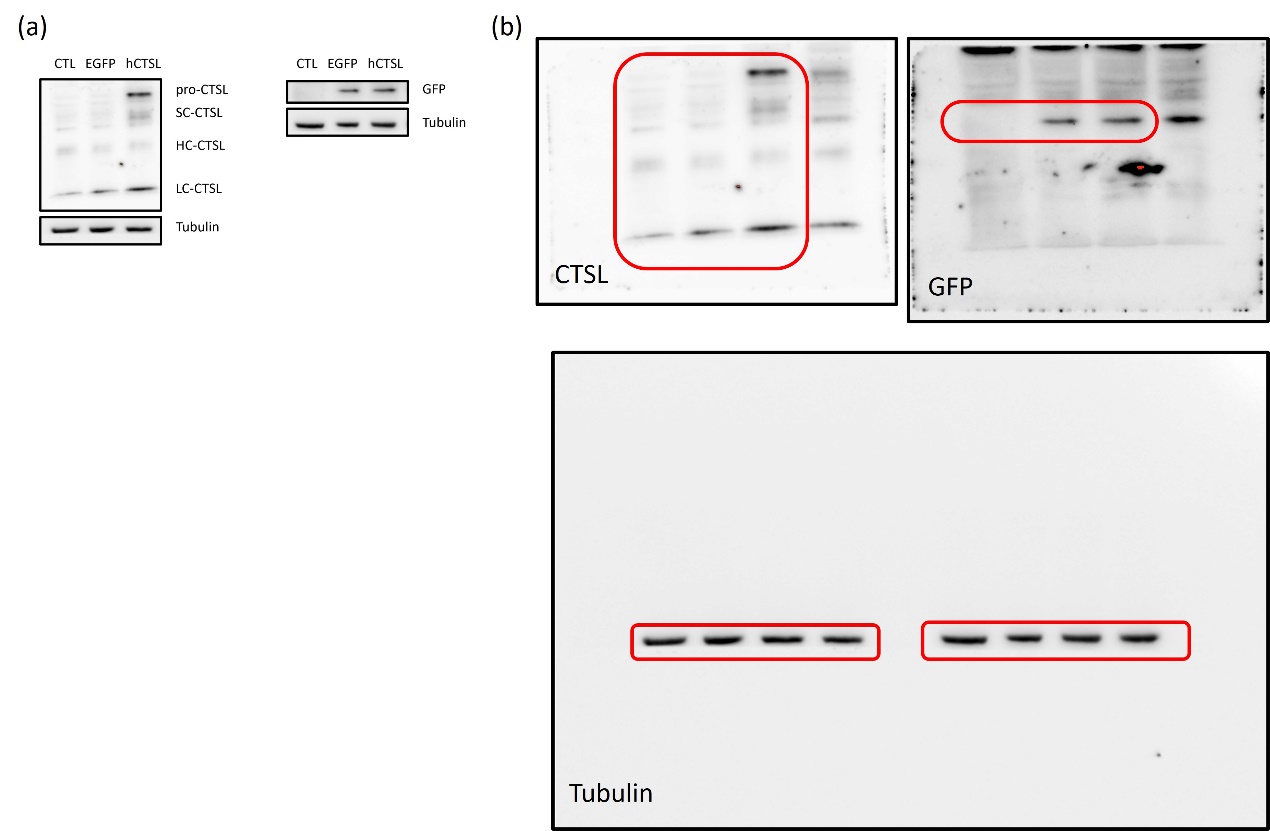
**

**Figure S11. The original Western blotting results for CTSL/Tubulin and GFP/Tubulin analysis.** (A) The original cropped Western blotting results as shown in Figure 8c in the manuscript. (B) The original full-length Western blots for the analyses on CTSL and Tubulin.

**Figure S12**

**
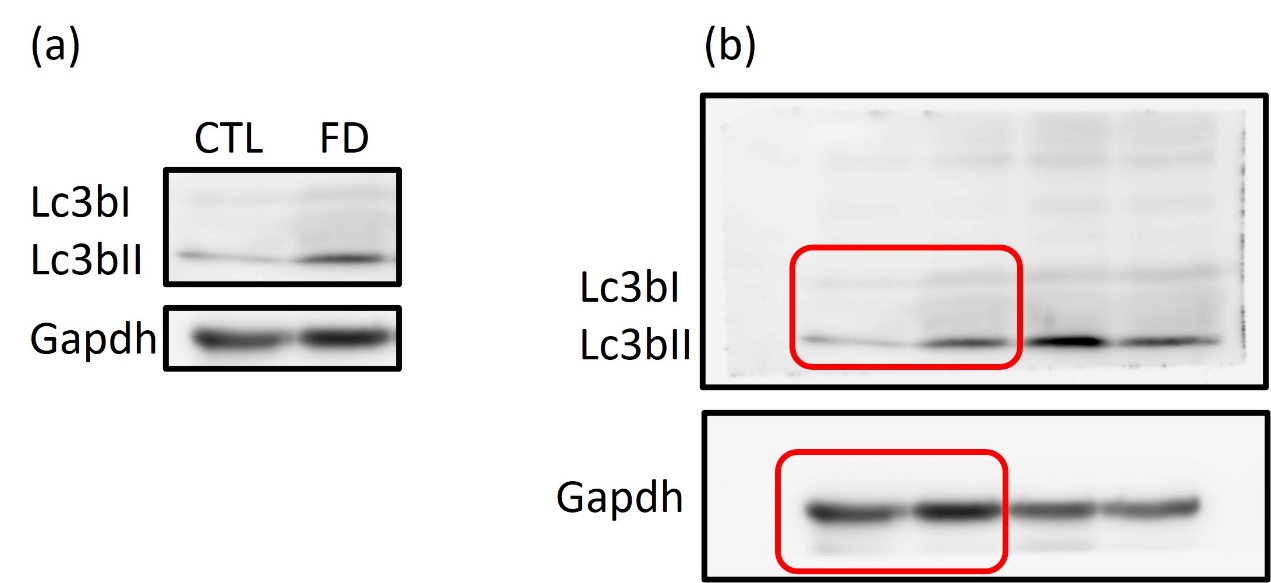
**

**Figure S12. The original Western blotting results for Lc3b/Gapdh analysis.** (A) The original cropped Western blotting results as shown in Figure 10a in the manuscript. (B) The original full-length Western blots for the analyses on Lc3b and Gapdh.

**Figure S13**

**
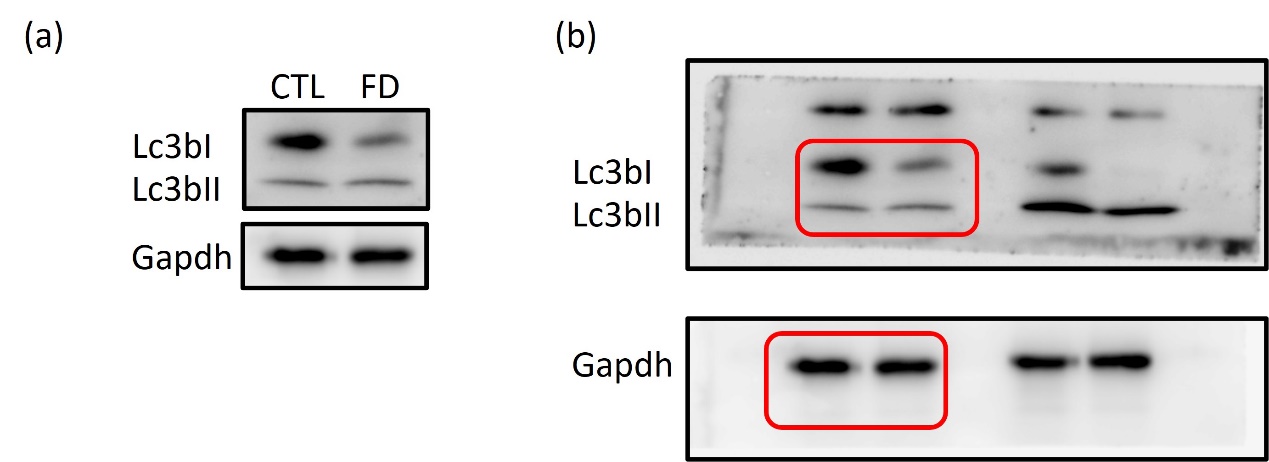
**

**Figure S13. The original Western blotting results for Lc3b/Gapdh analysis.** (A) The original cropped Western blotting results as shown in Figure 10b in the manuscript. (B) The original full-length Western blots for the analyses on Lc3b and Gapdh.

**Figure S14**

**
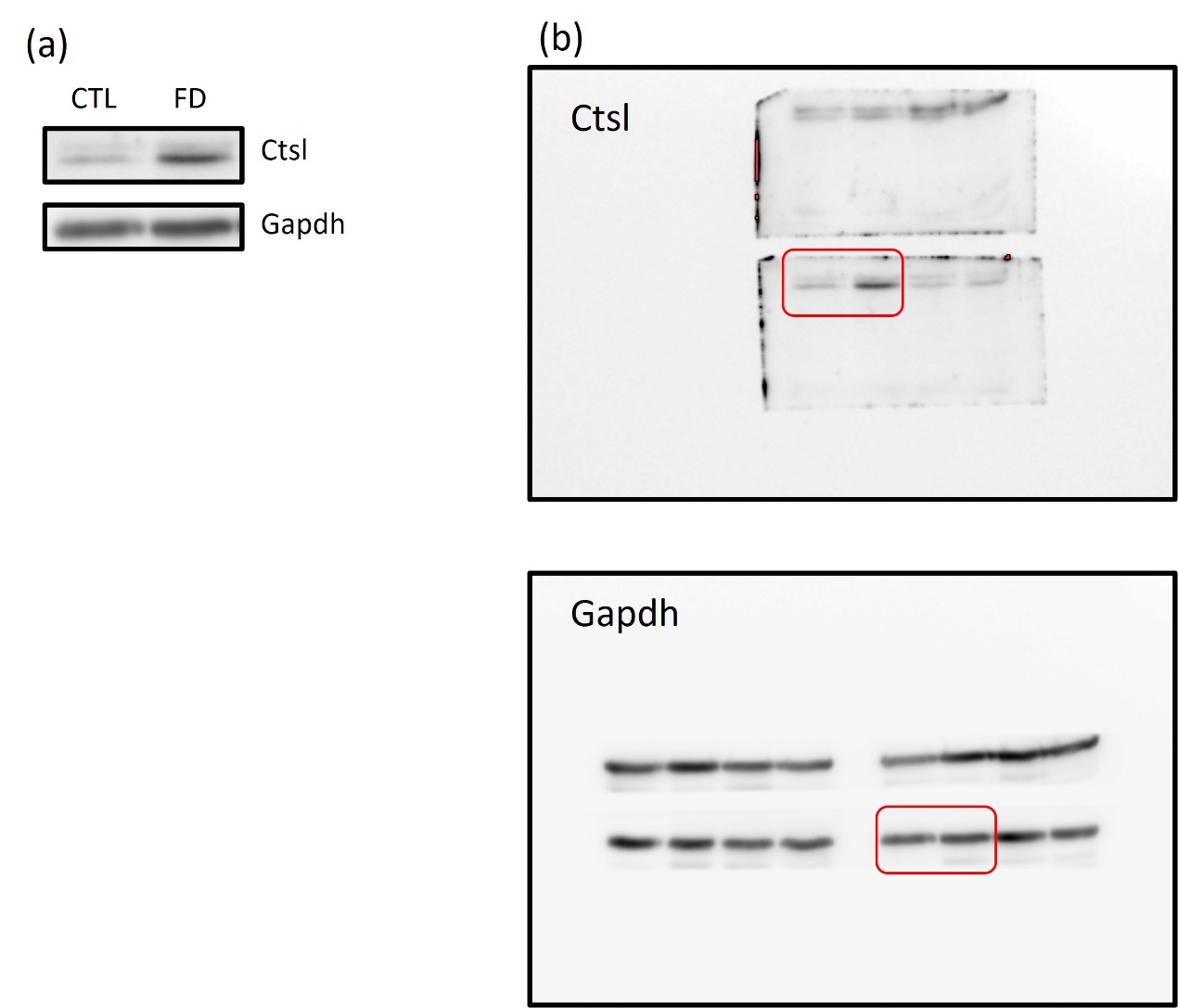
**

**Figure S14. The original Western blotting results for Ctsl/Gapdh analysis.** (A) The original cropped Western blotting results as shown in Figure 10e in the manuscript. (B) The original full-length Western blots for the analyses on ctsl and Gapdh.

**Figure S15**

**
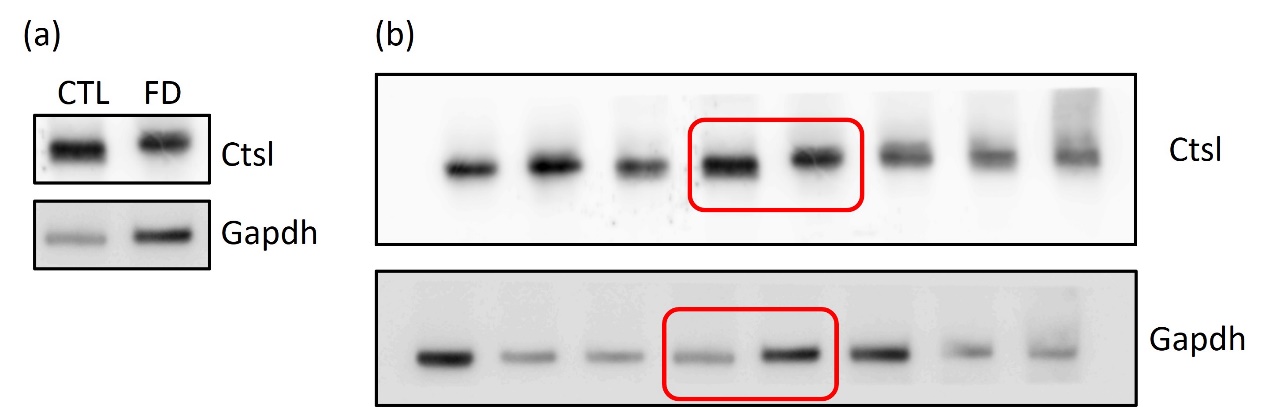
**

**Figure S15. The original Western blotting results for Ctsl/Gapdh analysis.** (A) The original cropped Western blotting results as shown in Figure 10g in the manuscript. (B) The original full-length Western blots for the analyses on ctsl and Gapdh.
