# Supplementary figures and images for: Disturbed intracellular folate homeostasis impairs autophagic flux and increases hepatocytic lipid accumulation
Source: BMC Biol. 2024 Jul 2;22:146. doi: 10.1186/s12915-024-01946-6 (PMC11220954; doi:10.1186/s12915-024-01946-6)

**Table 1**


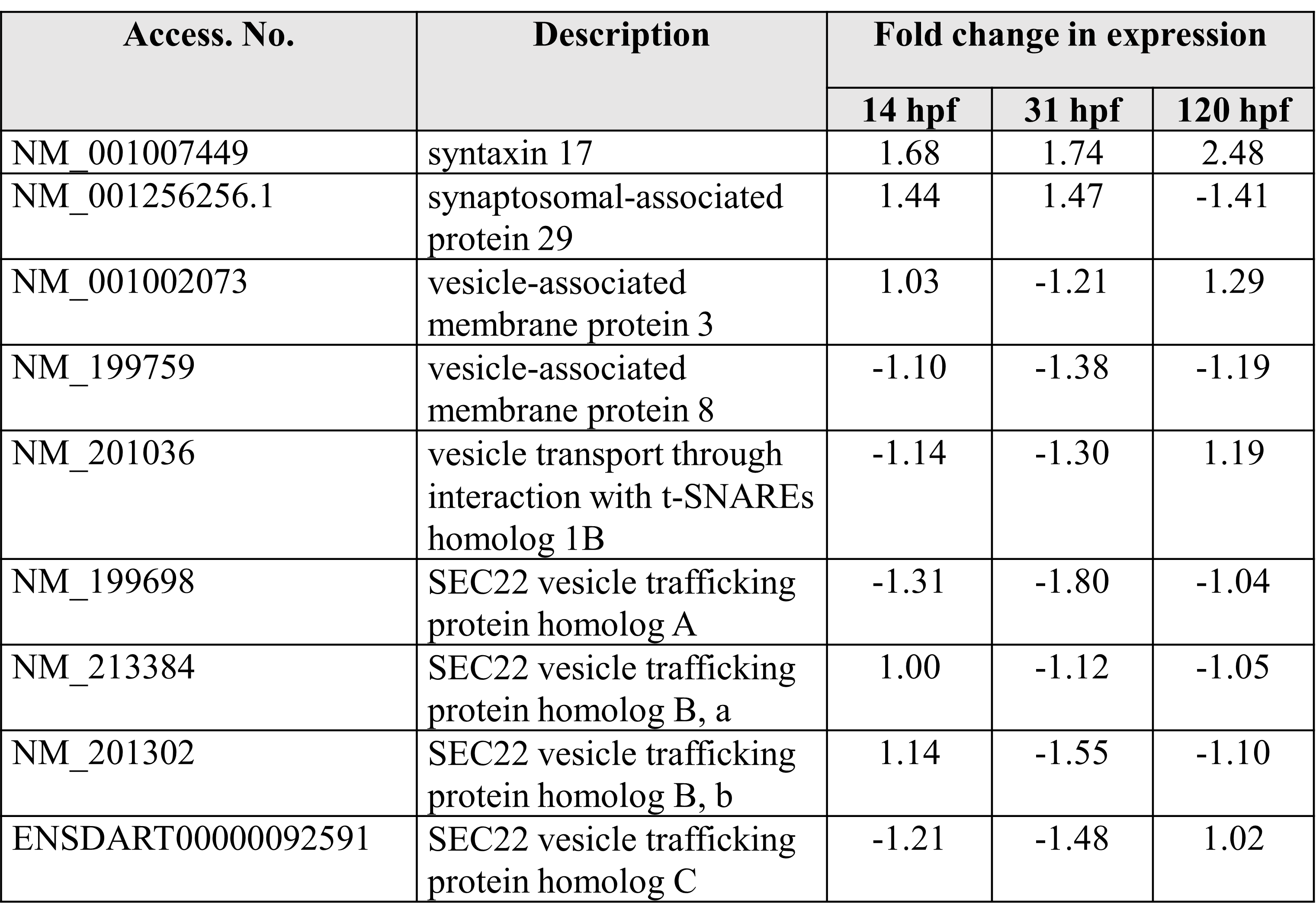

Supplement: Supplementary file 5 — Additional file 5: Table 1. The changes in expression levels of genes related to autolysosome maturation, as determined by microarray data. [file 12915_2024_1946_MOESM5_ESM.docx]
